# Supplementary material for: Targeting SOX10-deficient cells to reduce the dormant-invasive phenotype state in melanoma
Source: Nat Commun. 2022 Mar 16;13:1381. doi: 10.1038/s41467-022-28801-y (PMC8927161; doi:10.1038/s41467-022-28801-y)

Supplementary Materials to Targeting SOX10-deficient cells to reduce the dormant-invasive phenotype state in melanoma

Supplementary Methods

Cell culture: WM1366 and WM1361 cells were kindly donated by Dr. Meenhard Herlyn (The Wistar Institute, Philadelphia, PA, in 2005). Short-tandem repeat analysis was completed for WM1366 and WM1361 in September 2020 and August 2020, respectively.

Inhibitors**:** GDC-0152 and LCL161 were purchased from Selleck Chemicals.

Western blotting primary antibodies**:** ERBB3 (#4754, 1:1000), PDGFRβ (#3169, 1:1000), SOX10 (#89356, 1:1000), MITF (#12590, 1:1000), pS780-Rb (#9307, 1:1000), $p21^{Cip1}$ (#2947, 1:1000), ZEB1 (#3396S, 1:1000), WNT5 (#2530, 1:1000), N-cadherin (#13116, 1:1000), HSP90 (#4877, 1:3000), pRB S807/811 (#9308, 1:1000), cIAP1 (#7065, 1:1000), cIAP2 (#3130, 1:500), and XIAP (#2045, 1:1000) antibodies were purchased from Cell Signaling Technology. FN1 (ab45688, 1:1000) antibody was purchased from Abcam (Cambridge, MA). Actin (A2066, 1:2000) antibody was purchased from Sigma-Aldrich Co. Cyclin D3 (sc-182, 1:500) antibody was purchased from Santa Cruz Biotechnology. $p27^{Kip1}$ (#610241, 1:1000) and pFAK Y397 (#611806, 1:1000) antibodies were purchased from BD Transduction Laboratory.

Western blotting secondary antibodies**:** Goat Anti-Mouse IgG (#401215, 1:4000) and Goat Anti-Rabbit IgG (#401315, 1:4000) were purchased from Sigma-Aldrich Co.

Immunohistochemistry**:** SOX10 antigen retrieval was performed on the Ventana Discovery ULTRA staining platform using Discovery CCI (Ventana cat#950-500) for 52 minutes. Primary

immunostaining was performed using rabbit SOX10 antibody (Abcam [SP267], ab227680, 1:200) and horseradish peroxidase (HRP) multimer cocktail secondary antibodies (Ventana cat#760-500). Immune complexes were visualized using the UltraView Universal DAB (diaminobenzidine tetrahydrochloride) Detection Kit (Ventana cat#760-500). Slides were washed with a Tris-based reaction buffer (Ventana cat# 950-300) and stained with hematoxylin II (Ventana cat #790-2208) for 8 minutes.

MeWo, MeWo #2.1 and MeWo #4.11 xenografts were obtained from mice at 35 days post-injection. Tissues were fixed in formalin and paraffin embedded. Paraffin slides were deparaffinized in Shandon Varistain Gemini ES Autostainer. Antigen retrieval was performed with DAKO PTLink using Citrate Buffer (pH 6.0) at 98˚C for 20 minutes. Primary immunostaining was performed using antibodies against Ki67 (Abcam, ab16667, 1:200), $p21^{Cip1}$ (Cell Signaling Tech, #2947,1:200), and (Abcam [SP267], ab227680, 1:200) for 30 minutes at room temperature. Biotinylated anti-Rabbit (Vector Labs, cat#: BA-1000, 1:200) secondary antibodies and ABC-HRP complexes (Vector Labs, cat#: PK6100) were applied for 30 minute incubations each at room temperature. Three TBS-tween washes were performed between each step above. The signals were visualized using DAB substrate (DAKO, cat# K3468). Slides were washed with deionized water and then counterstained with hematoxylin.

TCGA mRNA correlation analyses: The cBioPortal (v3.7.22) for Cancer Genomics [1,2] website was used to perform correlation analysis between SOX10 and BIRC3 for TCGA SKCM RNA-seq expression data [3].

RNA-seq: Raw FASTQ sequencing reads for six cell lines (MM001, MM011, MM031, MM057, MM074, MM087) with SOX10 knockdown and control samples as well as a second dataset with A375 shSOX10 and parental samples were obtained from the Sequence Read Archive under the accession numbers SRP215051 and SRP029434, respectively, using the SRA toolkit (v 2.10.4)

[4]. Raw FASTQ RNA sequencing reads for M1, M2, M3 and M4 untreated mouse cell lines and 53 cutaneous melanoma cell lines were obtained from the SRA under the accession number SRP247646 and SRP074198, respectively. Melanoma cell state data were gathered from Tsoi et al [5]. For parental and PLX8394-resistant 1205Lu cell line samples, 200ng aliquot of each sample was transferred into library preparation which uses an automated variant of the Illumina TruSeq™ Stranded mRNA Sample Preparation Kit. The final libraries were sequenced on Illumina NovaSeq 6000 using 101 bp paired-end with an eight-base index barcode read. Raw FASTQ sequencing reads were mapped against the Homo sapiens (GRCh38.p12) or murine (GRCm38.p6) reference genomes. Further information was utilized from the gene transfer format annotation by GENCODE (v28, v30 or M25) using RSEM (v1.2.28) [6]. Total read counts and normalized Transcripts Per Million (TPM) were obtained using RSEM's calculate-expression function. Differential gene expression analysis was performed using the DESeq2 (v1.28.1) [7] package. A paired-sample comparison model was used for analysis of the Wouters [8] dataset. GSEA was performed to identify significantly altered pathways [9,10]. Single sample GSEA (ssGSEA) [11] gene set scores were calculated for each sample using the GSVA package (v1.40.1) [12] in R (v4.0.2 https://www.R-project.org/).

Statistical analysis: For IncuCyte experiments comparing treatments within the same cell line, growth was calculated as area under the curve (AUC), each treatment was normalized to the untreated control, and statistical analysis was performed using Student's two sample *t*-tests, assuming unequal variance.

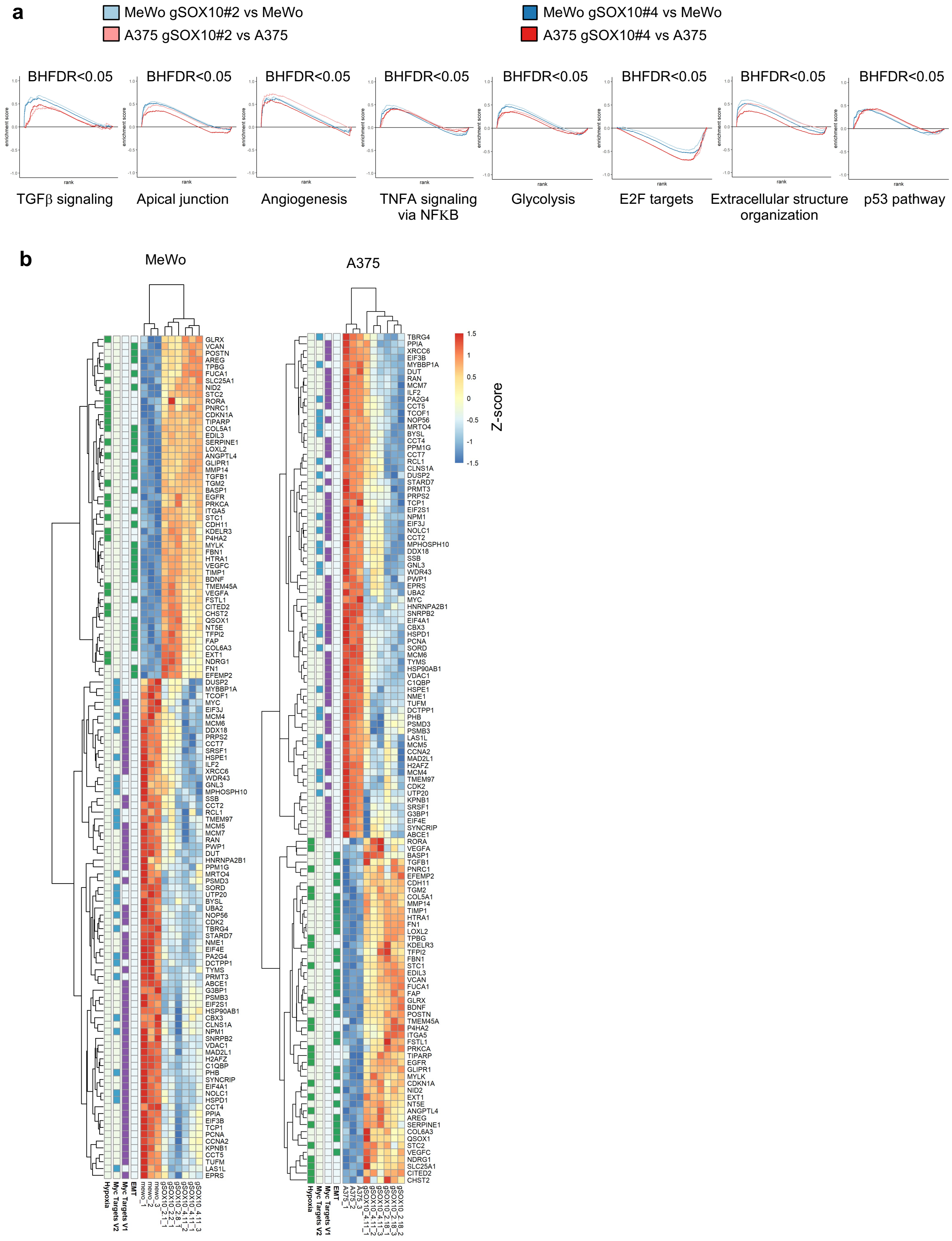
a
MeWo gSOX10#2 vs MeWo
A375 gSOX10#2 vs A375
MeWo gSOX10#4 vs MeWo
A375 gSOX10#4 vs A375
BHFDR<0.05
TGFβ signaling
Apical junction
Angiogenesis
TNFA signaling via NFKB
Glycolysis
E2F targets
Extracellular structure organization
p53 pathway
enrichment score
rank
b
MeWo
A375
Z-score
Hypoxia
Myc Targets V2
Myc Targets V1
EMT

Supplementary Figure Legends

Supplementary Figure 1. Characterization of the SOX10-regulated transcriptome.

A. Enrichment plots of TGFβ signaling, Apical Junction, Angiogenesis, TNFA signaling via NFκB, Glycolysis, E2F targets, Extracellular structure organization and p53 pathway comparing SOX10 knockout (guide#2 and guide#4) cells with parental cells. BHFDR as indicated. **B.** Heatmaps showing z-score values from A375 (left panel) and MeWo (right panel) RNA-seq data for genes that are commonly enriched in all four gSOX10 vs parental comparisons for the gene sets shown in Figure 2C (EMT, Hypoxia, MYC-1 targets, MYC-2 targets), as indicated in left-most columns for each heatmap.

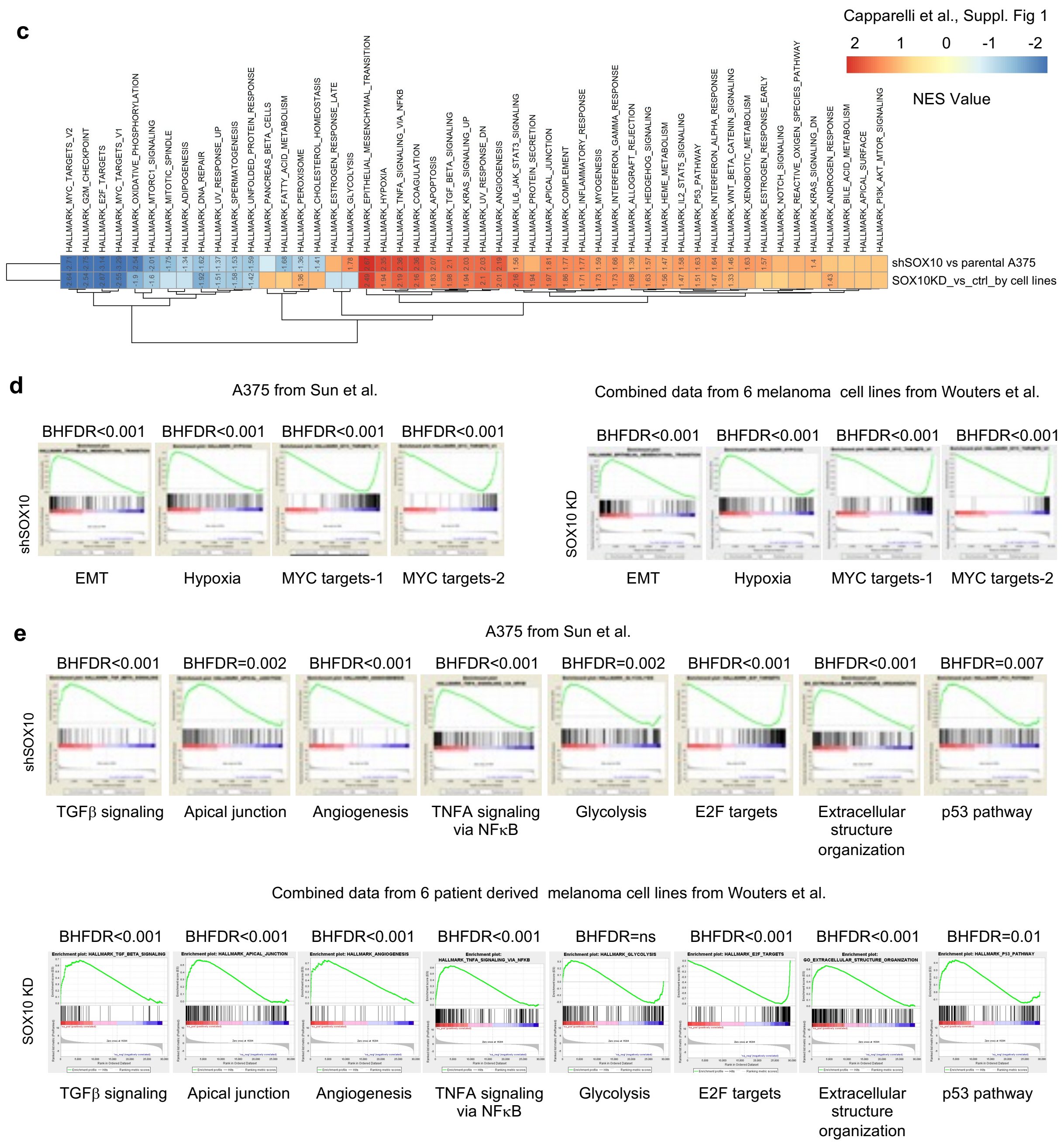

Capparelli et al., Suppl. Fig 1
c
2 1 0 -1 -2
NES Value
HALLMARK_MYC_TARGETS_V2
HALLMARK_G2M_CHECKPOINT
HALLMARK_E2F_TARGETS
HALLMARK_MYC_TARGETS_V1
HALLMARK_OXIDATIVE_PHOSPHORYLATION
HALLMARK_MTORC1_SIGNALING
HALLMARK_MITOTIC_SPINDLE
HALLMARK_ADIPOGENESIS
HALLMARK_DNA_REPAIR
HALLMARK_UV_RESPONSE_UP
HALLMARK_SPERMATOGENESIS
HALLMARK_UNFOLDED_PROTEIN_RESPONSE
HALLMARK_PANCREAS_BETA_CELLS
HALLMARK_FATTY_ACID_METABOLISM
HALLMARK_PEROXISOME
HALLMARK_CHOLESTEROL_HOMEOSTASIS
HALLMARK_ESTROGEN_RESPONSE_LATE
HALLMARK_GLYCOLYSIS
HALLMARK_EPITHELIAL_MESENCHYMAL_TRANSITION
HALLMARK_HYPOXIA
HALLMARK_TNFA_SIGNALING_VIA_NFKB
HALLMARK_COAGULATION
HALLMARK_APOPTOSIS
HALLMARK_TGF_BETA_SIGNALING
HALLMARK_KRAS_SIGNALING_UP
HALLMARK_UV_RESPONSE_DN
HALLMARK_ANGIOGENESIS
HALLMARK_IL6_JAK_STAT3_SIGNALING
HALLMARK_PROTEIN_SECRETION
HALLMARK_APICAL_JUNCTION
HALLMARK_COMPLEMENT
HALLMARK_INFLAMMATORY_RESPONSE
HALLMARK_MYOGENESIS
HALLMARK_INTERFERON_GAMMA_RESPONSE
HALLMARK_ALLOGRAFT_REJECTION
HALLMARK_HEDGEHOG_SIGNALING
HALLMARK_HEME_METABOLISM
HALLMARK_IL2_STAT5_SIGNALING
HALLMARK_P53_PATHWAY
HALLMARK_INTERFERON_ALPHA_RESPONSE
HALLMARK_WNT_BETA_CATENIN_SIGNALING
HALLMARK_XENOBIOTIC_METABOLISM
HALLMARK_ESTROGEN_RESPONSE_EARLY
HALLMARK_NOTCH_SIGNALING
HALLMARK_REACTIVE_OXIGEN_SPECIES_PATHWAY
HALLMARK_KRAS_SIGNALING_DN
HALLMARK_ANDROGEN_RESPONSE
HALLMARK_BILE_ACID_METABOLISM
HALLMARK_APICAL_SURFACE
HALLMARK_PI3K_AKT_MTOR_SIGNALING
shSOX10 vs parental A375
SOX10KD_vs_ctrl_by cell lines
d
A375 from Sun et al.
Combined data from 6 melanoma cell lines from Wouters et al.
BHFDR<0.001
BHFDR<0.001
BHFDR<0.001
BHFDR<0.001
shSOX10
EMT
Hypoxia
MYC targets-1
MYC targets-2
BHFDR<0.001
BHFDR<0.001
BHFDR<0.001
BHFDR<0.001
SOX10 KD
EMT
Hypoxia
MYC targets-1
MYC targets-2
e
A375 from Sun et al.
BHFDR<0.001
BHFDR=0.002
BHFDR<0.001
BHFDR<0.001
BHFDR=0.002
BHFDR<0.001
BHFDR<0.001
BHFDR=0.007
shSOX10
TGFβ signaling
Apical junction
Angiogenesis
TNFA signaling via NFκB
Glycolysis
E2F targets
Extracellular structure organization
p53 pathway
Combined data from 6 patient derived melanoma cell lines from Wouters et al.
BHFDR<0.001
BHFDR<0.001
BHFDR<0.001
BHFDR<0.001
BHFDR=ns
BHFDR<0.001
BHFDR<0.001
BHFDR=0.01
SOX10 KD
TGFβ signaling
Apical junction
Angiogenesis
TNFA signaling via NFκB
Glycolysis
E2F targets
Extracellular structure organization
p53 pathway

Supplementary Figure 1. Characterization of the SOX10-regulated transcriptome.

C. Heatmap showing GSEA normalized enrichment scores for the hallmark gene sets collection comparing A375 shSOX10 vs. control cells from the publicly available dataset [13] (upper row) and SOX10 KD vs. parental in six different patient-derived melanoma cells (MM001, MM011, MM031, MM057, MM074, MM087) from the publicly available dataset [8] (lower row). NES values are displayed for enriched gene sets (BHFDR <0.05). **D.** Enrichment plots of EMT, Hypoxia, MYC targets-1 and MYC targets-2 comparing A375 shSOX10 vs. A375 control cells from the publicly available dataset [13] (left) and SOX10 KD vs. parental in six different patient-derived melanoma cells (MM001, MM011, MM031, MM057, MM074, MM087) from the publicly available dataset [8] (right) BHFD<0.001. **E.** Enrichment plots as in A. comparing shSOX10 vs. parental A375 cells (top) and SOX10 KD vs. parental patient-derived melanoma cells (bottom). BHFDR as indicated.

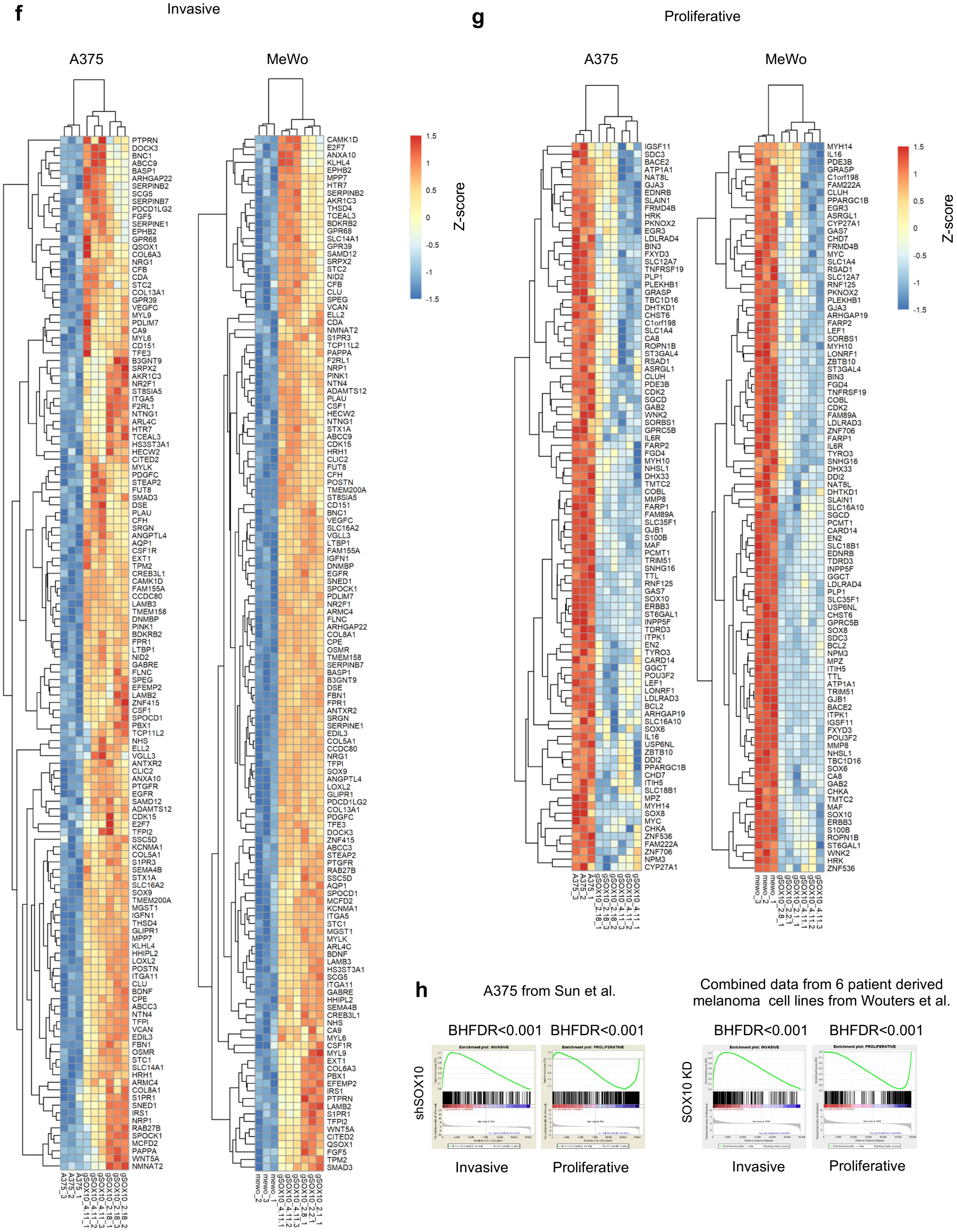
f
Invasive
A375
MeWo
Z-score
g
Proliferative
A375
MeWo
Z-score
h
A375 from Sun et al.
Combined data from 6 patient derived melanoma cell lines from Wouters et al.
BHFDR<0.001
BHFDR<0.001
BHFDR<0.001
BHFDR<0.001
shSOX10
SOX10 KD
Invasive
Proliferative
Invasive
Proliferative

Supplementary Figure 1. Characterization of the SOX10-regulated transcriptome.

F. Heatmaps showing z-score values for invasive gene set genes that are commonly enriched in all four gSOX10 vs parental comparisons from A375 (left panel) and MeWo (right panel panel), corresponding to Figure 2D (left panel). **G.** Heatmaps showing z-score values for proliferative gene set genes that are commonly enriched in all four gSOX10 vs parental comparisons from A375 (left panel) and MeWo (right panel), corresponding to Figure 2D (right panel). **H.** Enrichment plots of proliferative and invasive gene signatures [14] for the comparison of shSOX10 vs. parental A375 cells (left) and SOX10 KD vs. parental patient-derived melanoma cells (right). BHFDR<0.001. A paired-sample comparison model was used to analyze six SOX10 knockdowns vs. parental patient-derived melanoma cells data.

Capparelli et al., Suppl. Fig 2

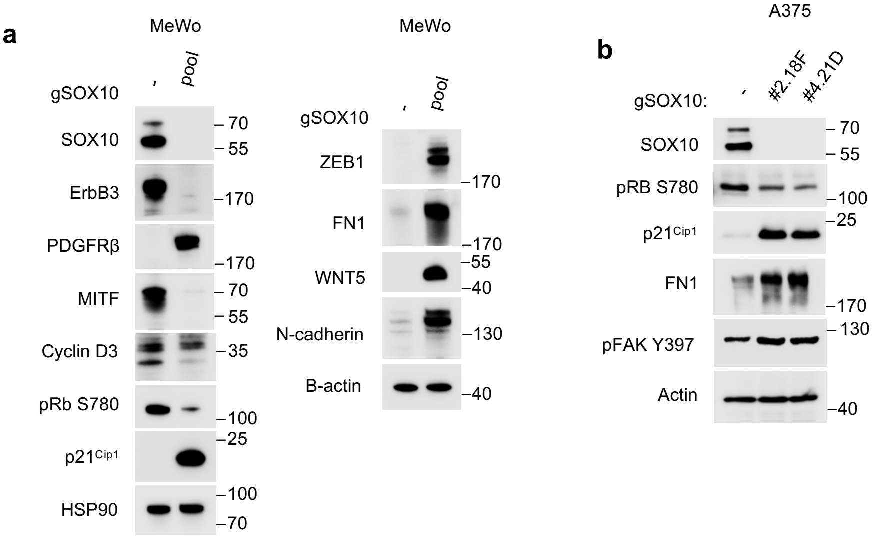

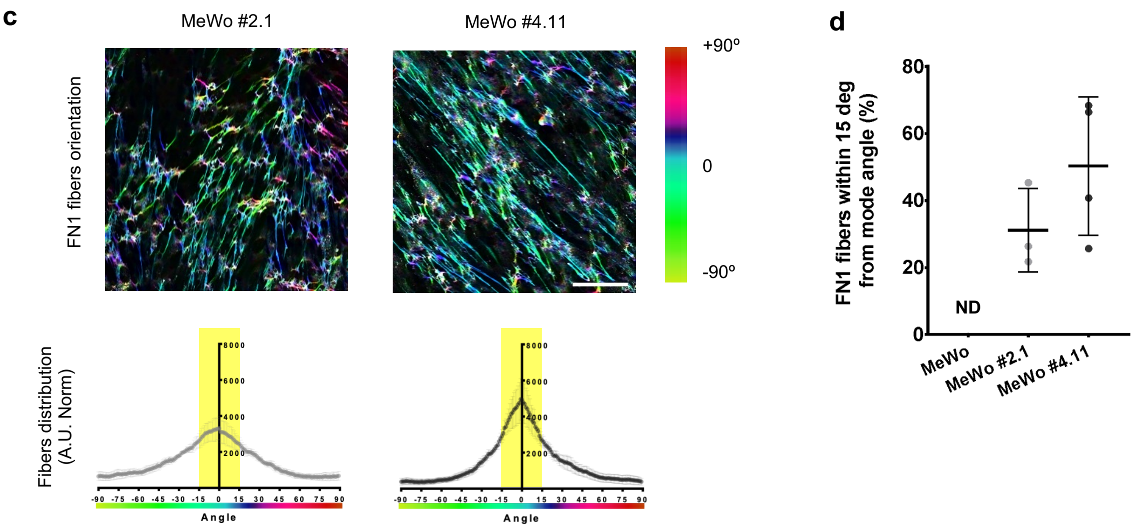

e Scratch Wound Assay statistic

Comparison	p-value	Mean Difference (effect size)	Confidence limits Lower 95%	Upper 95%
MeWo #2.1 vs MeWo (24h)	0.09016427	48.7821667	-5.6397469	103.20408
MeWo #2.1 vs MeWo (48h)	0.02810037	86.0946667	25.0102026	147.179131
MeWo #2.1 vs MeWo (72h)	0.04439204	114.965883	44.4651146	185.466652
MeWo #2.2 vs MeWo (24h)	0.10187653	101.537217	-15.777197	218.85163
MeWo #2.2 vs MeWo (48h)	0.02341348	117.336867	29.2135366	205.460197
MeWo #2.2 vs MeWo (72h)	0.03237171	121.998	17.2058971	226.790103
MeWo #2.8 vs MeWo (24h)	0.13974934	50.9984833	-25.343785	127.340752
MeWo #2.8 vs MeWo (48h)	0.03562519	77.5572667	12.111577	143.002956
MeWo #2.8 vs MeWo (72h)	0.01575488	136.557633	54.8676303	218.247636
MeWo #4.11 vs MeWo (24h)	0.03495172	242.252133	86.1726389	398.331628
MeWo #4.11 vs MeWo (48h)	0.00508367	347.697483	223.887156	471.50781
MeWo #4.11 vs MeWo (72h)	0.00090313	355.600967	274.793397	436.408536

Supplementary Figure 2. SOX10 regulates the expression of genes associated with a slow cycling and more invasive phenotype.

A. SOX10 knockout clones #2.1, # 2.2, # 2.8 and #4.11 were co-mixed at equal ratios (knockout pool). The same number of knockout pool and parental cells were seeded in six well plates. Cells were lysed and analyzed by Western blotting as indicated. The experiment was repeated independently twice with similar results. **B.** The same number of cells were seeded in six well plates for each cell line. Cells were lysed and analyzed by Western blotting, as indicated. The experiment was repeated independently twice with similar results. **C.** Representative images showing color-coded distribution of FN1 fibers detected from each cell line. Fibers within the range of cyan color depict those close to the mode angle orientation. The experiment was repeated independently twice with similar results, scale bar=50 μm. Below images, graphs show corresponding angle distribution curves, highlighting in yellow the fibers distributed within 15 degrees away from the mode angle. **D.** Quantification of S2C expressed as mean ± standard deviation. Note fibers were not detected (ND) in MeWo parental cells. **E.** Table showing statistical analysis comparing cell migration of MeWo parental and MeWo SOX10 knockout #2.1, #2.2, #2.8 and #4.11 cells at 24, 48 and 72 hours. p-values were calculated using two-sided t-test.

f

Spheroid (Core area)

		Confidence limits	
	% decrease	Lower 95%	Upper 95%
MeWo#2.1	0.91956585	0.88353394	0.94445031
MeWo#2.2	0.89079898	0.83406315	0.92813614
MeWo#2.8	0.93313945	0.81600176	0.97570448
MeWo#4.11	0.84632352	0.71150265	0.91813977

Spheroid (Area of outgrowth)

		Confidence limits	
	Fold change	Lower 95%	Upper 95%
MeWo#2.1	6.203081313	4.80744469	8.00388153
MeWo#2.2	4.630217701	2.36087742	9.08091024
MeWo#2.8	3.204240925	1.79641146	5.71537208
MeWo#4.11	9.329684282	4.51514433	19.2780125

Supplementary Figure 2. SOX10 regulates the expression of genes associated with a slow cycling and more invasive phenotype.

F. Tables showing statistical analysis comparing spheroid core area (upper table) and spheroid outgrowth (lower table) of MeWo parental and MeWo SOX10 knockout #2.1, #2.2, #2.8 and #4.11 cells at 24, 48 and 72 hours. p-values were calculated using two-sided one-sample t-test of the null hypothesis.

Capparelli et al., Suppl. Fig 3

a

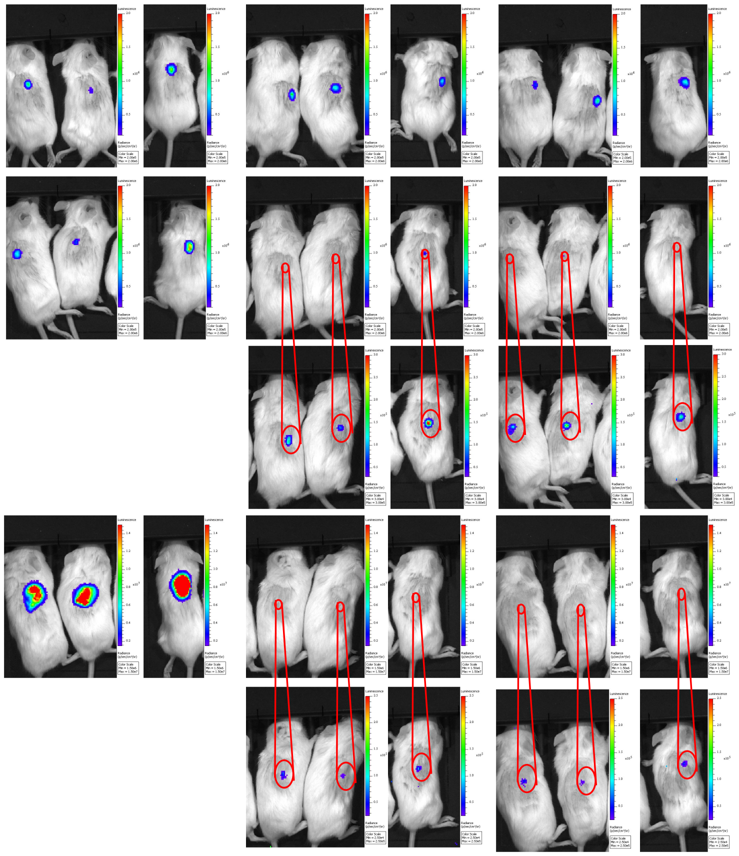

b

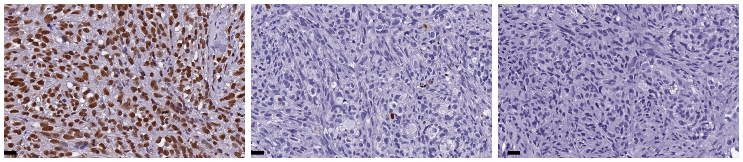

c

Ki67 IHC staining (% positive cells)

		Mean Difference	Confidence limits	
Comparison	p-value	(effect size)	Lower 95%	Upper 95%
MeWo vs MeWo SOX10 KO	0.00339299	41.5333333	30.3759691	52.6906975

p21 IHC staining (% positive cells)

		Mean Difference	Confidence limits	
Comparison	p-value	(effect size)	Lower 95%	Upper 95%
MeWo SOX10 KO vs MeWo	0.02148808	51.1833333	26.396352	75.9703147

d

Integrated second harmonic generation densities

	Effect size		Confidence limits	
Comparison	Location Shift	p-value	Lower 95%	Upper 95%
MeWo SOX10 vs Mewo	6.16 Norm A.U.	0.025	0.43	19.88

Supplementary Figure 3. SOX10 loss induces a quiescent/dormant like phenotype *in vivo*. **A.** 3 x 10^6 MeWo parental or CRISPR SOX10 knockout cells (#2.1 and #4.11) were injected intradermally into the backs of NOD.Cg-Prkdcscid Il2rgtm1Wjl/SzJ (NSG) mice. *In vivo* bioluminescence detection was conducted using the Caliper IVIS Lumina-XR System (Caliper Life Sciences), and data acquisition was conducted using LivingImage Version 4.0 software. The images show luciferase signal in MeWo parental and MeWo SOX10 knockout #2.1 and #4.11 at different time points, as indicated. **B.** IHC comparing SOX10 expression in MeWo parental versus SOX10 knockout (clones #2.1 and #4.11) tumors collected at the end of the experiment (Day 35). The staining was performed on three independent tumors generated either from parental or SOX10 knockout MeWo cells and representative images are shown. Scale bar, 20 μm. **C.** Tables showing statistical analysis comparing Ki67 IHC staining (% positive cells, upper table) and p21 IHC staining (% positive cells, lower table) of MeWo parental and MeWo SOX10 knockout tumors at day 35. p-values were calculated using two-sided t-test. **D.** Tables showing statistical analysis of second harmonic generation (SHG) of polarized light (ECM fibers orientation). p-values were calculated using Wilcoxon Two-Sample test and The Hodges-Lehmann estimate of location shift for the 95% confidence intervals.

a

Comparison	PGI ratio	Adjusted LL	Adjusted UL	Adjusted p-value
PGI_MeWo_GSK_dose50_21vsWT	3.24264	2.03521	5.16638	<.0001
PGI_MeWo_GSK_dose100_21vsWT	3.37966	2.12122	5.38470	<.0001
PGI_MeWo_GSK_dose50_411vsWT	3.34096	2.09693	5.32303	<.0001
PGI_MeWo_GSK_dose100_411vsWT	4.08894	2.56639	6.51476	<.0001

b

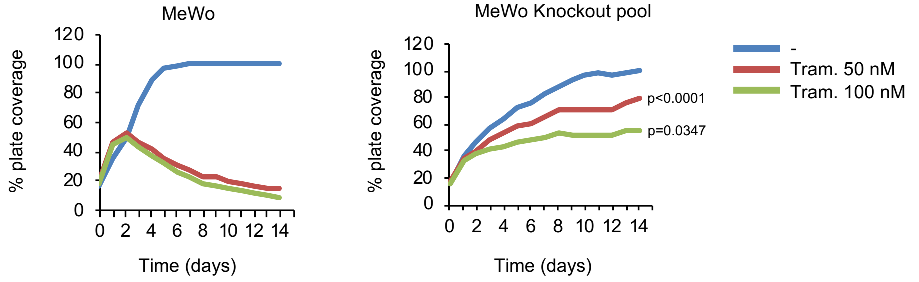

c

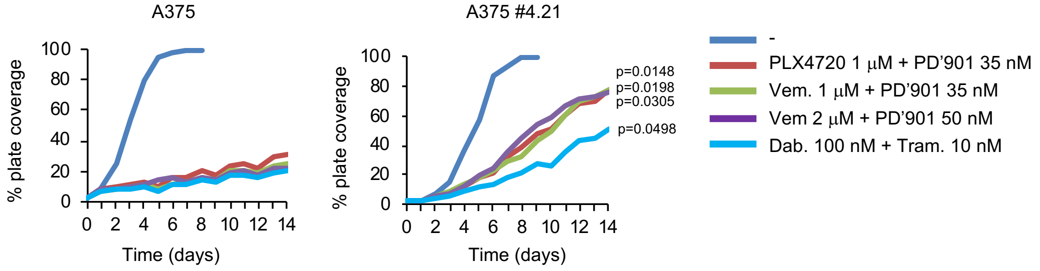

d

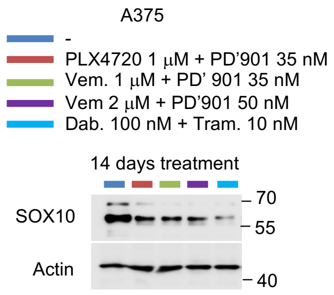

e

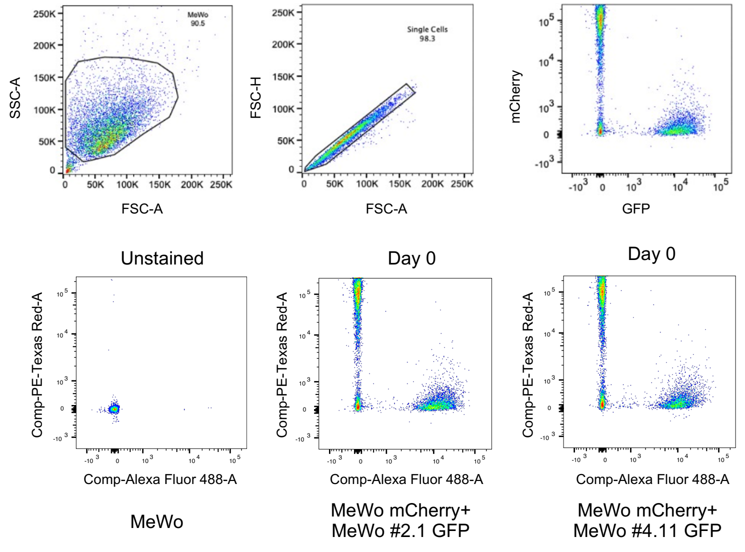

f

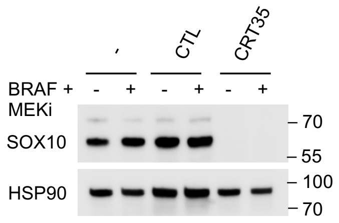

Supplementary Figure 4. SOX10 loss/downregulation in MAPK targeting agent resistant melanoma induces an invasive phenotype.

A. Statistical analysis for cell growth experiment shown in figure 5A. **B.** The same number of MeWo parental and SOX10 knockout pool cells treated with trametinib. Treatment was renewed 3 times per week. Shown is the mean from three independent experiments. p-values were calculated using two-sided t-test and represent statistical analysis between MeWo parental vs MeWo knockout pool at day 14. **C.** Cells were treated as indicated. Treatment was renewed 3 times per week. Shown is the mean from three independent experiments. p-values were calculated using two-sided t-test and represent statistical analysis between A375 parental vs A375 #4.21 at day 14. **D.** Cells were treated as in C. and lysates Western blotted as indicated. The experiment was repeated twice with similar results. **E.** mCherry-MeWo and GFP-MeWo SOX10 knockout #2.1 or #4.11 cells were co-mixed at the ratio of 2:1. The next day, cells were analyzed by FACS for mCherry and GFP positivity**.** The experiment was repeated independently twice with similar results. **F.** A375 parental, vehicle treated A375 xenograft derived cells (CTL) and CRT35 cells were analyzed by Western blotting. The experiment was repeated independently twice with similar results.

Capparelli et al., Suppl. Fig 4

g

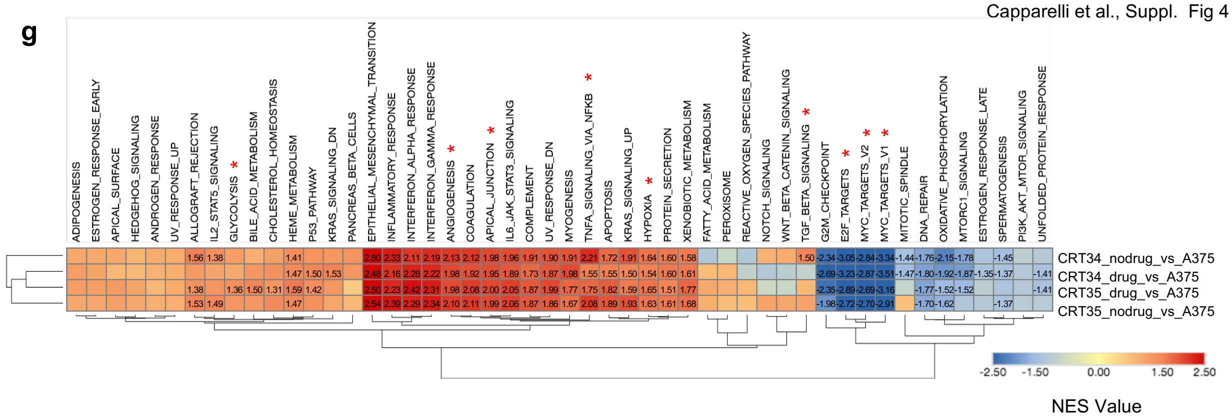

h

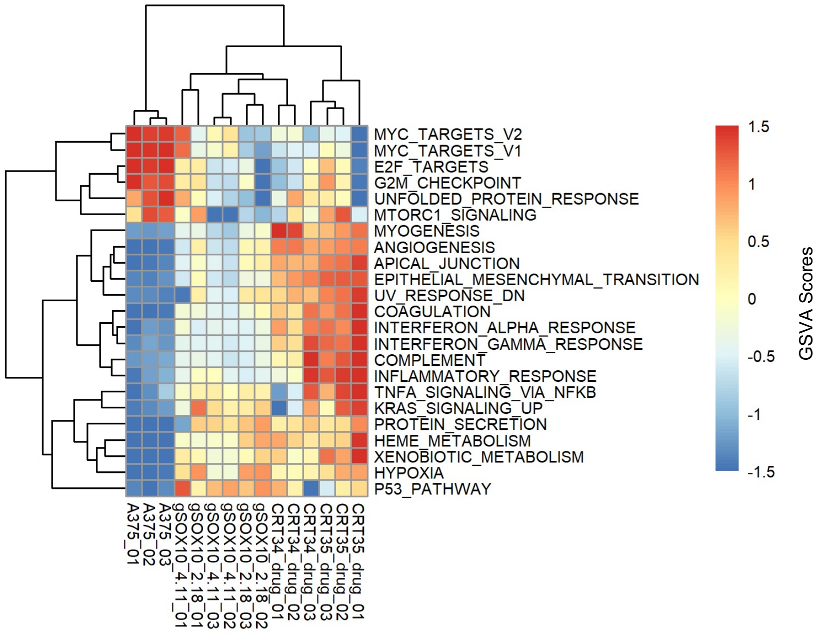

i

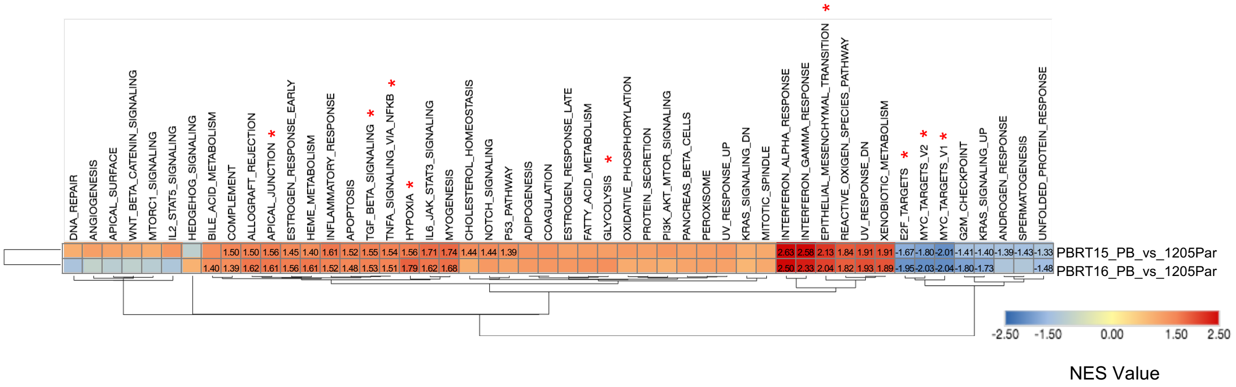

Supplementary Figure 4. SOX10 loss/downregulation in MAPK targeting agent resistant melanoma induces an invasive phenotype.

G. GSEA was used to perform pathway analysis between untreated A375 parental and each A375 tolerant/resistant (CRT34 and CRT35) cell line in the presence or absence of BRAFi+MEKi, separately. A heatmap showing unsupervised clustering of NES values for each comparison. NES values are displayed for significant gene sets (BHFDR < 0.05). **H.** A heat map showing single sample GSEA (ssGSEA) scores for commonly enriched genes in all CRT and gSOX10 A375 comparisons, corresponding to Fig 5E. **I.** GSEA pathway analysis between untreated 1205LuTR parental and each 1205LuTR tolerant/resistant (PBRT15 and PBRT16) cell line in the presence of 500 nM PLX8394, separately. A heatmap showing unsupervised clustering of NES values for each comparison. NES values are displayed for significant gene sets (BHFDR < 0.05).

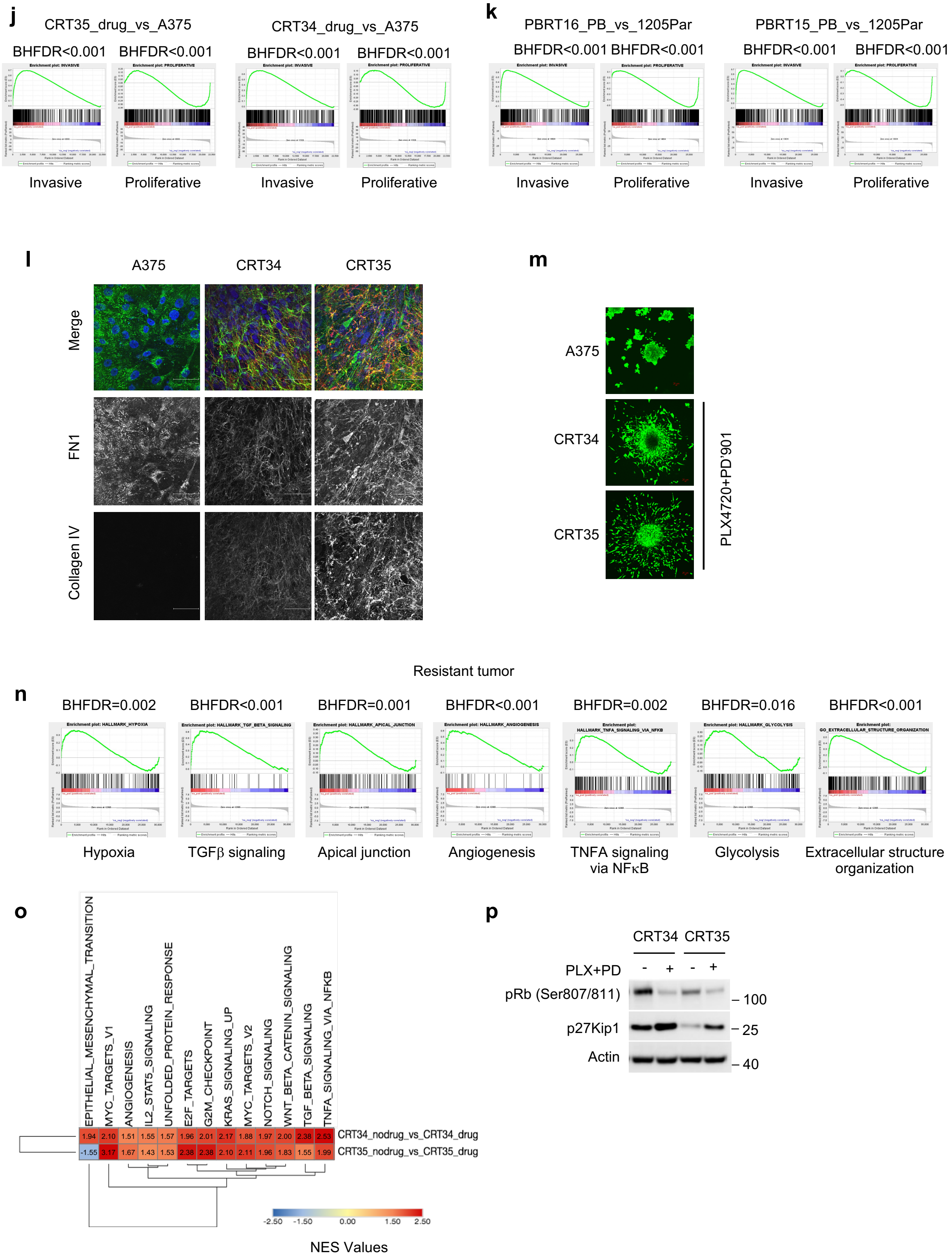
j
CRT35_drug_vs_A375
BHFDR<0.001 BHFDR<0.001
Invasive
Proliferative
CRT34_drug_vs_A375
BHFDR<0.001 BHFDR<0.001
Invasive
Proliferative
k
PBRT16_PB_vs_1205Par
BHFDR<0.001 BHFDR<0.001
Invasive
Proliferative
PBRT15_PB_vs_1205Par
BHFDR<0.001 BHFDR<0.001
Invasive
Proliferative
l
A375
CRT34
CRT35
Merge
FN1
Collagen IV
m
A375
CRT34
CRT35
PLX4720+PD'901
Resistant tumor
n
BHFDR=0.002
Hypoxia
BHFDR<0.001
TGFβ signaling
BHFDR=0.001
Apical junction
BHFDR<0.001
Angiogenesis
BHFDR=0.002
TNFA signaling via NFκB
BHFDR=0.016
Glycolysis
BHFDR<0.001
Extracellular structure organization
o
EPITHELIAL_MESENCHYMAL_TRANSITION
MYC_TARGETS_V1
ANGIOGENESIS
IL2_STAT5_SIGNALING
UNFOLDED_PROTEIN_RESPONSE
E2F_TARGETS
G2M_CHECKPOINT
KRAS_SIGNALING_UP
MYC_TARGETS_V2
NOTCH_SIGNALING
WNT_BETA_CATENIN_SIGNALING
TGF_BETA_SIGNALING
TNFA_SIGNALING_VIA_NFKB
CRT34_nodrug_vs_CRT34_drug
1.94 2.10 1.51 1.55 1.57 1.96 2.01 2.17 1.88 1.97 2.00 2.38 2.53
CRT35_nodrug_vs_CRT35_drug
-1.55 3.17 1.67 1.43 1.53 2.38 2.33 2.10 2.11 1.96 1.83 1.55 1.99
-2.50 -1.50 0.00 1.50 2.50
NES Values
p
CRT34 CRT35
PLX+PD - + - +
pRb (Ser807/811)
p27Kip1
Actin
100
25
40

Supplementary Figure 4. SOX10 loss/downregulation in MAPK targeting agent resistant melanoma induces an invasive phenotype.

J. Enrichment plots of proliferative and invasive gene signature [14] for tolerant/resistant (CRT35 and CRT34) cells in the presence of 1 µM PLX4720 plus 35 nM PD0325901, versus parental A375 cells. BHFDR<0.001. **K.** Enrichment plots as in J. [14] for tolerant/resistant (PBRT15 and PBRT16) cells in the presence of PLX8394 500 nM, versus parental 1205LuTR cells. BHFDR<0.001. **L.** Cells were stained for FN1 and collagen IV. The experiment was performed twice, and representative images are shown. Scale bars, 50 µm. **M.** Spheroids in 3D collagen comparing parental A375 (basal condition), CRT34 and CRT35 (treated with BRAFi+MEKi). The experiment was performed independently three times, and representative images are shown, scale bar, 25 µm. **N.** Enrichment plots of Hypoxia, TGFβ signaling, Apical Junction, Angiogenesis, TNFA signaling via NFκB Extracellular, Glycolysis and Extracellular structure organization comparing patient samples before and after MAPK targeting agent treatment [13]. BHFDR as indicated. **O.** GSEA pathway analysis between tolerant/resistant (CRT34 and CRT35) A375 cell lines in the absence or presence of BRAFi+MEKi. A heatmap showing unsupervised clustering of NES values for gene sets identified as significant (BHFDR <0.05) in both comparisons. **P.** Cells were plated and treated with BRAFi+MEKi for 24 hours. Cells were lysed and Western blotted with the antibodies indicated. Experiment was repeated independently twice with similar results.

a

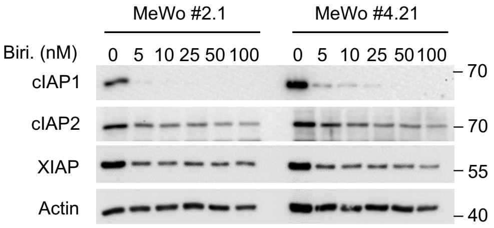

b

Comparison	PGI ratio	Adjusted LL	Adjusted UL	Adjusted p-value
PGI_MeWo_Biri_dose5_21vsWT	0.48944	0.24201	0.98986	0.0265
PGI_MeWo_Biri_dose10_21vsWT	0.43757	0.18909	1.01257	0.0284
PGI_MeWo_Biri_dose25_21vsWT	0.41515	0.20527	0.83960	0.0039
PGI_MeWo_Biri_dose50_21vsWT	0.36860	0.18226	0.74545	0.0007
PGI_MeWo_Biri_dose100_21vsWT	0.33426	0.16528	0.67601	0.0002
PGI_MeWo_Biri_dose5_411_WT	0.77836	0.38487	1.57417	0.3144
PGI_MeWo_Biri_dose10_411vsWT	0.69620	0.34424	1.40799	0.2929
PGI_MeWo_Biri_dose25_411vsWT	0.58668	0.29009	1.18651	0.0988
PGI_MeWo_Biri_dose50_411vsWT	0.50942	0.25189	1.03025	0.0286
PGI_MeWo_Biri_dose100_411vsWT	0.45428	0.22463	0.91875	0.0119

c

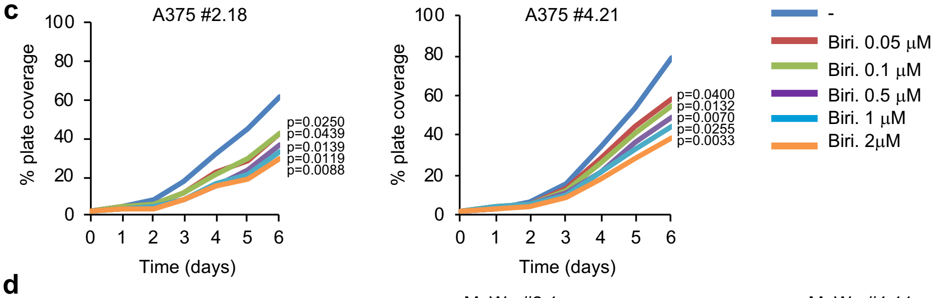

d

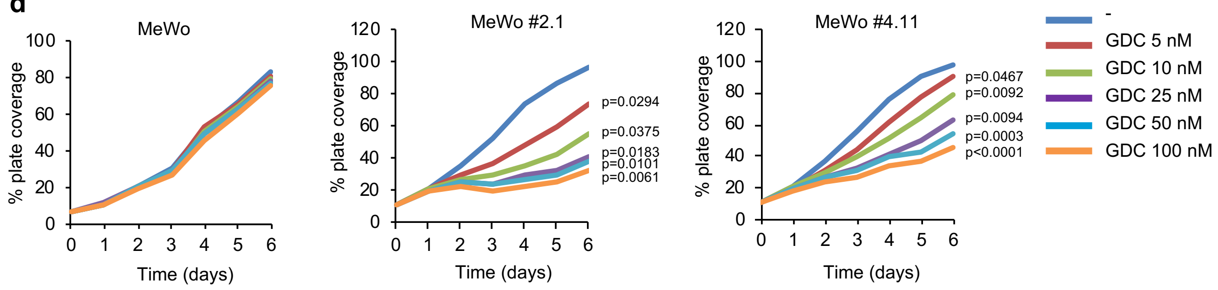

e

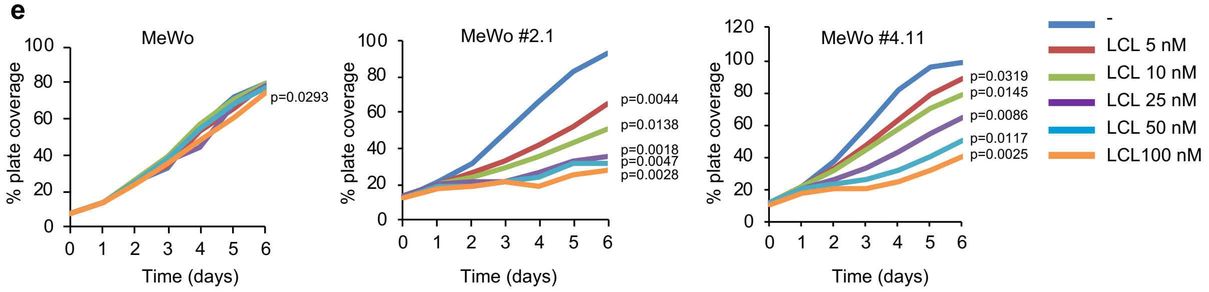

f

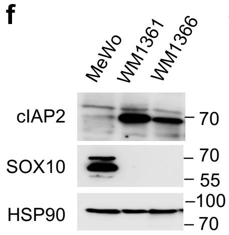

g

Zero-inflation model for BIRC3

Gene	Odds Ratio	95%CL	95%CL	p-value
BIRC3	1.91	1.08	1.66	<.0001

Mean model for BIRC3

Gene	Mean diff (*)	95%CL	95%CL	p-value
BIRC3	-0.16	-0.25	-0.06	0.002

Supplementary Figure 5. Synthetic lethality of IAP inhibitors towards SOX10 deficient cells. **A.** Cells were treated with birinapant (0, 5, 10, 25, 50 and 100 nM) for 24 hours, lysed and Western blotted as indicated. The experiment was repeated twice with similar results. **B.** Statistical analysis for cell growth experiment shown in figure 6B. **C.** Birinapant dose-response growth curves in A375 #2.18 and A375 #4.21 cells. Cells were treated with birinapant (0, 5, 10, 25, 50 and 100 nM). Treatment was renewed 3 times per week. For statistical analysis, cell growth was calculated as AUC and each treatment was normalized to the untreated control within each experimental replicate. Shown is the mean from three independent experiments. p-values were calculated using two-sided t-test and represent statistical analysis within the same cell line. **D.** GDC-0152 dose-response growth curves in MeWo parental and SOX10 knockout cells #2.1 and #4.11. Cells were treated with GDC-0152 (0, 5, 10, 25, 50 and 100 nM). Treatment was renewed 3 times per week. Statistical analysis as in C. Shown is the mean from three independent experiments. p-values were calculated using two-sided t-test and represent statistical analysis within the same cell line. **E.** Cells were treated as in D. except LCL161 was used. Statistical analysis as in C. Shown is the mean from three independent experiments. p-values were calculated using two-sided t-test and represent statistical analysis within the same cell line **F.** Cell lysates were Western blottied, as indicated. The experiment was repeated independently twice with similar results. **G.** Separately zero-inflated negative binomial (ZINB) regression models for single cell RNA-seq counts of BIRC3 as dependent on SOX10 counts and the total RNA-seq counts per cell as an exposure.

h

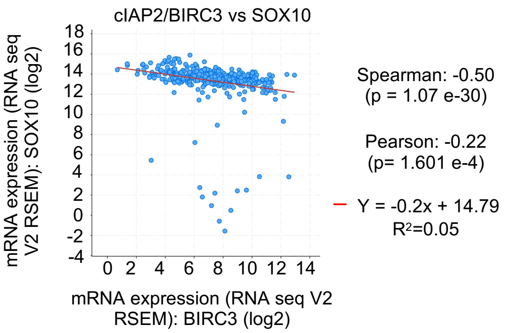

i

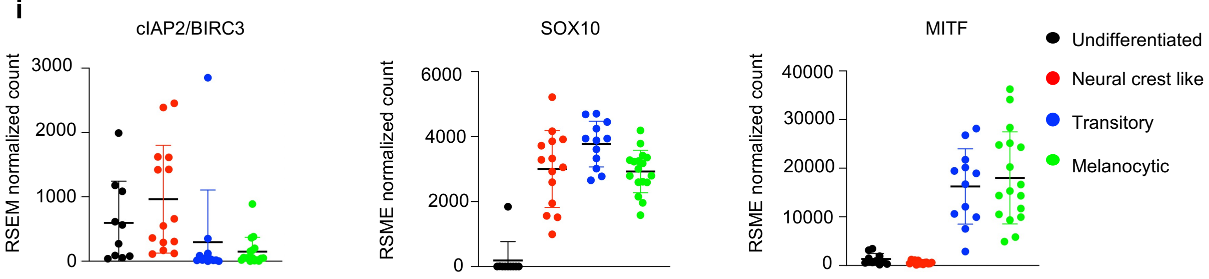

Group 1	Group 2	P-value
Undifferentiated	Neural crest like	0.39810274
Undifferentiated	Transitory	0.232904187
Undifferentiated	Melanocytic	0.010893525
Neural crest like	Transitory	0.02871392
Neural crest like	Melanocytic	0.000157
Transitory	Melanocytic	0.181499

Group 1	Group 2	P-value
Undifferentiated	Neural crest like	1.99E-06
Undifferentiated	Transitory	4.58E-07
Undifferentiated	Melanocytic	1.04E-06
Neural crest like	Transitory	0.627443562
Neural crest like	Melanocytic	0.951346
Transitory	Melanocytic	0.572214

Group 1	Group 2	P-value
Undifferentiated	Neural crest like	0.002401472
Undifferentiated	Transitory	1.44E-20
Undifferentiated	Melanocytic	2.52E-25
Neural crest like	Transitory	2.00E-40
Neural crest like	Melanocytic	1.34E-50
Transitory	Melanocytic	0.664373

j

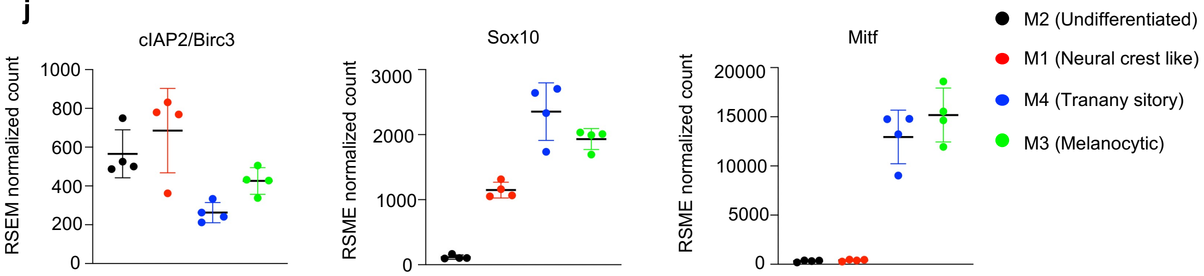

Group 1	Group 2	P-value
M2	M1	0.262340406
M2	M4	9.35E-06
M2	M3	0.10102274
M1	M4	3.10E-08
M1	M3	0.00592381
M4	M3	0.00540339

Group 1	Group 2	P-value
M2	M1	1.67E-68
M2	M4	4.25E-117
M2	M3	1.91E-102
M1	M4	3.20E-08
M1	M3	6.03E-05
M4	M3	0.12879065

Group 1	Group 2	P-value
M2	M1	0.164965096
M2	M4	2.59E-106
M2	M3	1.94E-115
M1	M4	2.88E-93
M1	M3	8.19E-102
M4	M3	0.34320751

k

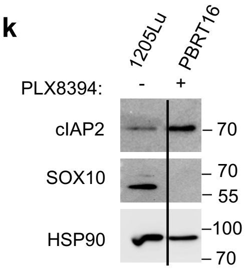

Supplementary Figure 5. Synthetic lethality of IAP inhibitors towards SOX10 deficient cells. **H.** Correlation analysis of RNA level expression of cIAP2/BIRC3 and SOX10 within the TCGA dataset. **I.** Box plots showing cIAP2/BIRC3, SOX10, and MITF normalized RSEM values across undifferentiated (n=10), neural crest-like (n=14), transitory (n=12) and melanocytic (n=17) human melanoma cell lines samples separated by defined cell states [5]. Shown is the mean ± SD. DESeq2 was used to perform pair-wise comparisons between each state for all genes, and p-values for select genes are reported. **J.** Box plots showing cIAP2/Birc3, Sox10, and Mitf normalized RSEM values across M1 (n=4), M2 (n=4), M3 (n=4) and M4 (n=4) mouse melanoma cell lines samples separated by defined states [15]. Shown is the mean ± SD. DESeq2 was used to perform pair-wise comparisons between each state for all genes, and p-values for select genes are reported. **K.** Cells were treated with 500 nM PLX8394 for 24 hours, lysed and Western blotted as indicated. The experiment was repeated independently twice with similar results.

l

CRT35

Comparison	PGI ratio	Adjusted LL	Adjusted UL	Adjusted p-value
PGI_dose005_CRT35vsA375	0.84	0.42	1.65	0.499
PGI_dose01_CRT35vsA375	0.59	0.30	0.94	0.087
PGI_dose05_CRT35vsA375	**0.47**	**0.24**	**0.86**	**0.014**
PGI_dose1_CRT35vsA375	**0.34**	**0.17**	**0.67**	**< 0.001**
PGI_dose2_CRT35vsA375	**0.31**	**0.16**	**0.61**	**< 0.001**

m

CRT34

Comparison	PGI ratio	Adjusted LL	Adjusted UL	Adjusted p-value
PGI_dose005_CRT34vsA375	0.66	0.25	1.69	0.163
PGI_dose01_CRT34vsA375	0.47	0.18	1.22	**0.029**
PGI_dose05_CRT34vsA375	**0.33**	**0.13**	**0.86**	**0.001**
PGI_dose1_CRT34vsA375	**0.31**	**0.12**	**0.80**	**0.001**
PGI_dose2_CRT34vsA375	**0.33**	**0.13**	**0.86**	**0.001**

n

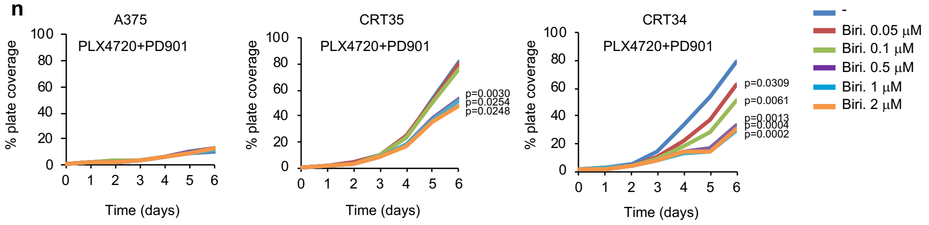

o

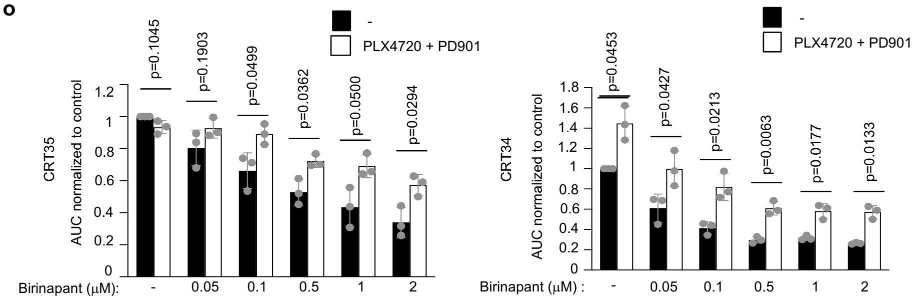

p

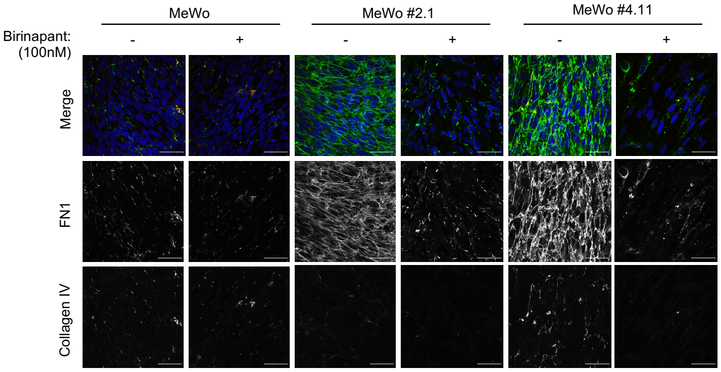

Supplementary Figure 5. Synthetic lethality of IAP inhibitors towards SOX10 deficient cells. **L., M.** Statistical analysis for cell growth experiment shown in figure 6F. **N.** Cells were treated birinapant (0, 0.1, 0.5, 1 and 2 μM) in the presence of BRAFi+MEKi. Treatment was renewed 3 times per week. Statistical analysis as in C. p-values represent statistical analysis within the same cell line. **O.** Quantification of the growth assay in N. Shown in mean ± SD from 3 independent experiments. p-values were calculated using two-sided t-test. **P.** Cells were treated 100nM birinapant for 6 days. Treatment was renewed every 48 hours. Cells were stained for FN1 and collagen IV. The experiment was performed twice, and representative images are shown. Scale bars, 50μm.

q

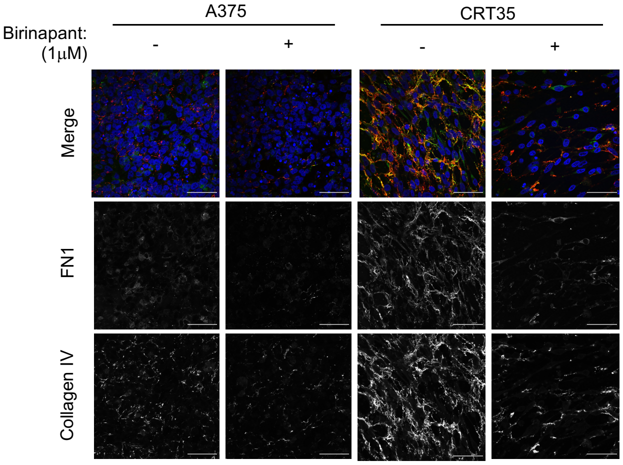

r

Estimated Median Survival Time and the corresponding 95% Confidence Interval (CI).

Tx	Median Time to Tumor Regrow (days)	95% CI
BRAF/MEKi	35.0	(24.0, 56.0)
BRAFi/MEKi/Biri	84.0	(63.0, infinity)

s

Tx	Median survival (in days)	95% CI	p - value*	p - value**
**Control**	10.0	(10.0, 14.0)	<.001	-
**Birinipant**	14.0	(14.0, 17.0)		0.023
**BRAF/MEki**	59.0	(45.0, 87.0)		<.001
**BRAF/Meki/Biri**	112.0	(94.0, -)		<.001

*p-value from global Log-rank test
**p-value from Log-rank test comparing Control vs. each of 3 Tx group

Pairwise comparison by Log-rank Test and the corresponding False Discovery Rate (FDR) adjusted p-values.

Comparison	False Discovery Rate p-value
**Control vs. Birinipant**	0.023
**Control vs. BRAF/MEKi**	<.001
**Control vs. BRAF/MEKi/Biri**	<.001
**Birinipant vs. BRAF/MEKi**	<.001
**Birinipant vs. BRAF/MEKi/Biri**	<.001
**BRAF/MEKi vs. BRAF/MEKi/Biri**	0.019

t

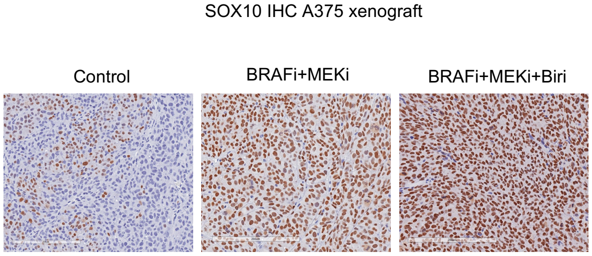

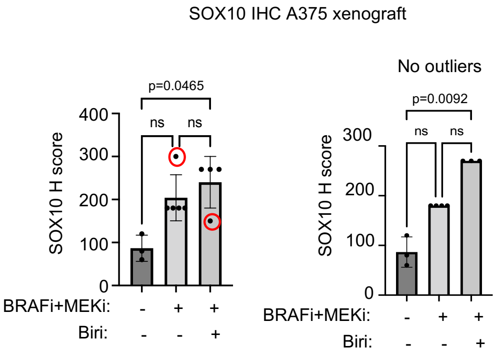

Supplementary Figure 5. Synthetic lethality of IAP inhibitors towards SOX10 deficient cells.

Q. Cells were treated with 1μM birinapant for 6 days. Treatment was renewed every 48 hours. Cells were stained for FN1 and collagen IV. The experiment was performed twice, and representative images are shown. Scale bars, 50μm. **R.** Table with Estimated Median Time to Tumor Regrowth (tumor volume > 100mm^3) and the corresponding 95% Confidence Interval (CI). **S.** Table with Estimated median survival and the corresponding 95% Confidence Interval (CI) and p-values for the mouse survival curve for the *in vivo* experiment shown in figure 6G and 6H. p-values were calculated using the two-sided log-rank tests and were adjusted for multiple testing to control for the False Discovery Rate (FDR) using the method of Benjamini and Hochberg. **T.** IHC comparing SOX10 expression in A375 xenograft tumor collected at the end of the experiment as indicated in Fig 6G. Mice were treated with BRAF + MEK inhibitors (200 PPM PLX4720 plus 7PPM PLX2695) and/or injected intraperitoneally, twice per week, with 100 μL birinapant solution (3 mg/ml). Scale bar, 20 μm. H-score analysis was performed as described in the Materials and Methods by Dr. Xu, who had knowledge of the sample's condition but was blinded to the expected result. Shown is the mean ± SD of 3, 5 or 4 independent tumors for control, BRAFi/MEKi and BRAFi/MEKi/biri, respectively. p-values were calculated using The Kruskall-Wallis test and multiple comparisons were done using a Dunn's correction. Values circle in red represent outliers, which were calculated using the ROUT method with Q = 1%.

Supplementary References

1 Gao, J. *et al.* Integrative analysis of complex cancer genomics and clinical profiles using the cBioPortal. *Sci Signal* **6**, pl1, doi:10.1126/scisignal.2004088 (2013).

2 Cerami, E. *et al.* The cBio cancer genomics portal: an open platform for exploring multidimensional cancer genomics data. *Cancer Discov* **2**, 401-404, doi:10.1158/2159-8290.CD-12-0095 (2012).

3 Hoadley, K. A. *et al.* Cell-of-Origin Patterns Dominate the Molecular Classification of 10,000 Tumors from 33 Types of Cancer. *Cell* **173**, 291-304 e296, doi:10.1016/j.cell.2018.03.022 (2018).

4 Leinonen, R., Sugawara, H., Shumway, M. & International Nucleotide Sequence Database, C. The sequence read archive. *Nucleic Acids Res* **39**, D19-21, doi:10.1093/nar/gkq1019 (2011).

5 Tsoi, J. *et al.* Multi-stage Differentiation Defines Melanoma Subtypes with Differential Vulnerability to Drug-Induced Iron-Dependent Oxidative Stress. *Cancer Cell* **33**, 890-904 e895, doi:10.1016/j.ccell.2018.03.017 (2018).

6 Li, B. & Dewey, C. N. RSEM: accurate transcript quantification from RNA-Seq data with or without a reference genome. *BMC Bioinformatics* **12**, 323, doi:10.1186/1471-2105-12-323 (2011).

7 Love, M. I., Huber, W. & Anders, S. Moderated estimation of fold change and dispersion for RNA-seq data with DESeq2. *Genome Biol* **15**, 550, doi:10.1186/s13059-014-0550-8 (2014).

8 Wouters, J. *et al.* Robust gene expression programs underlie recurrent cell states and phenotype switching in melanoma. *Nat Cell Biol* **22**, 986-998, doi:10.1038/s41556-020-0547-3 (2020).

9 Mootha, V. K. *et al.* PGC-1alpha-responsive genes involved in oxidative phosphorylation are coordinately downregulated in human diabetes. *Nat Genet* **34**, 267-273, doi:10.1038/ng1180 (2003).

10 Subramanian, A. *et al.* Gene set enrichment analysis: a knowledge-based approach for interpreting genome-wide expression profiles. *Proc Natl Acad Sci U S A* **102**, 15545-15550, doi:10.1073/pnas.0506580102 (2005).

11 Barbie, D. A. *et al.* Systematic RNA interference reveals that oncogenic KRAS-driven cancers require TBK1. *Nature* **462**, 108-112, doi:10.1038/nature08460 (2009).

12 Hanzelmann, S., Castelo, R. & Guinney, J. GSVA: gene set variation analysis for microarray and RNA-seq data. *BMC Bioinformatics* **14**, doi:10.1186/1471-2105-14-7 (2013).

13 Sun, C. *et al.* Reversible and adaptive resistance to BRAF(V600E) inhibition in melanoma. *Nature* **508**, 118-122, doi:nature13121 [pii]10.1038/nature13121 (2014).

14 Verfaillie, A. *et al.* Decoding the regulatory landscape of melanoma reveals TEADS as regulators of the invasive cell state. *Nat Commun* **6**, 6683, doi:10.1038/ncomms7683 (2015).

15 Perez-Guijarro, E. *et al.* Multimodel preclinical platform predicts clinical response of melanoma to immunotherapy. *Nat Med* **26**, 781-791, doi:10.1038/s41591-020-0818-3 (2020).

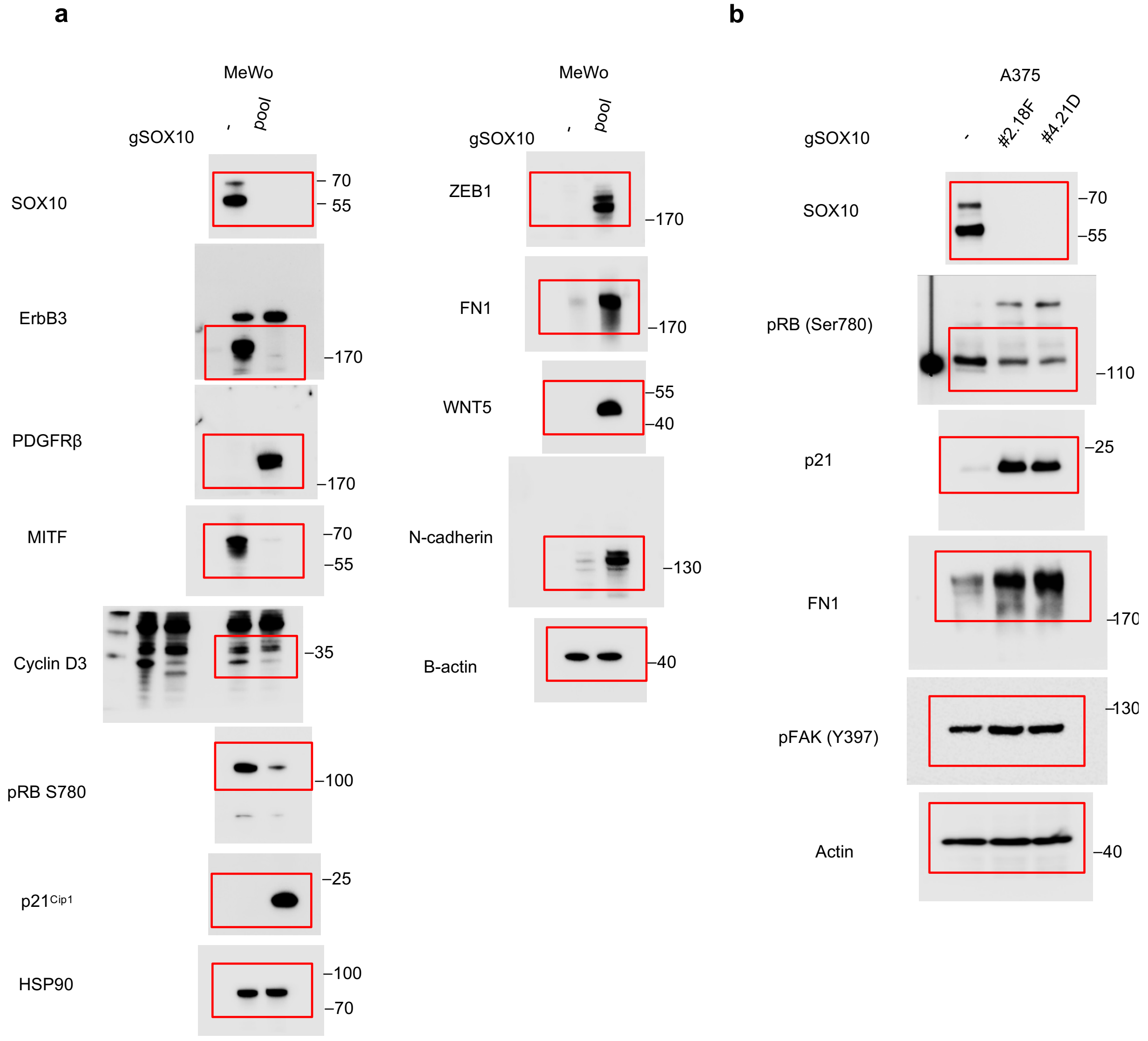
a
MeWo
gSOX10
-
pool
SOX10
70
55
ErbB3
170
PDGFRβ
170
MITF
70
55
Cyclin D3
35
pRB S780
100
p21Cip1
25
HSP90
100
70
MeWo
gSOX10
-
pool
ZEB1
170
FN1
170
WNT5
55
40
N-cadherin
130
B-actin
40
b
A375
gSOX10
-
#2.18F
#4.21D
SOX10
70
55
pRB (Ser780)
110
p21
25
FN1
170
pFAK (Y397)
130
Actin
40

d

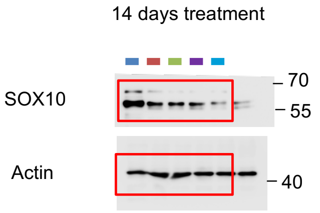
14 days treatment
SOX10
70
55
Actin
40

f

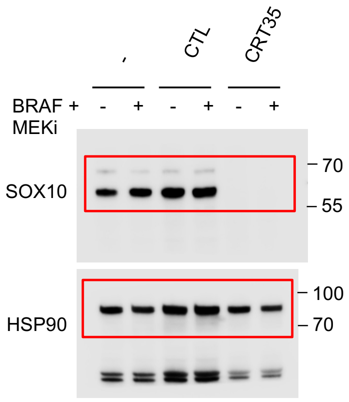
CTL
CRT35
BRAF + MEKi
- + - + - +
SOX10
70
55
HSP90
100
70

o

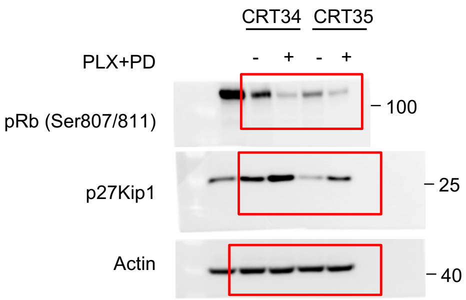

e

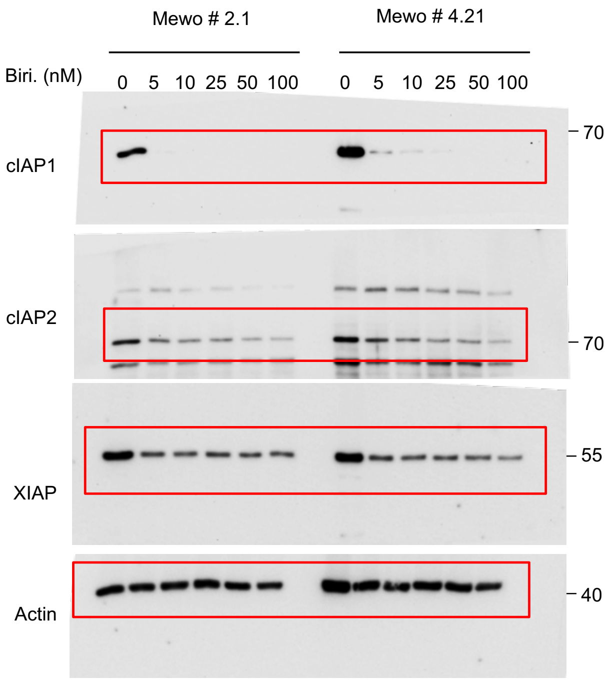
Mewo # 2.1
Mewo # 4.21
Biri. (nM)
0 5 10 25 50 100
0 5 10 25 50 100
cIAP1
cIAP2
XIAP
Actin
70
70
55
40

f

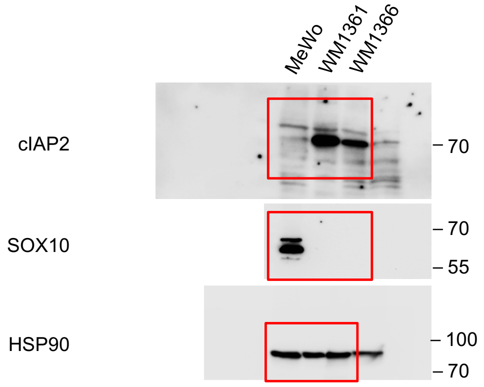

i

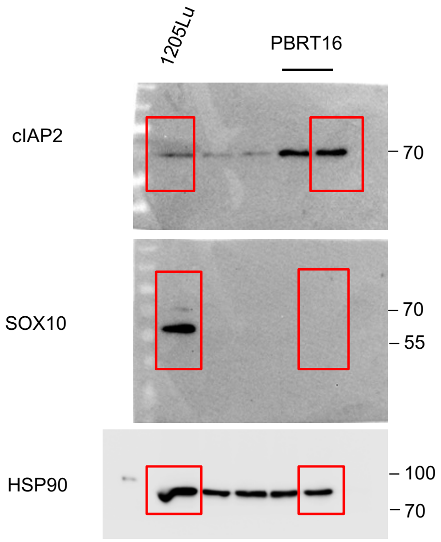

Supplement: Supplementary file 1 — Supplementary Information [file 41467_2022_28801_MOESM1_ESM.pdf]
