# Supplementary material for: Targeting SOX10-deficient cells to reduce the dormant-invasive phenotype state in melanoma
Source: Nat Commun. 2022 Mar 16;13:1381. doi: 10.1038/s41467-022-28801-y (PMC8927161; doi:10.1038/s41467-022-28801-y)
Supplement: Supplementary file 3 — Reporting Summary [file 41467_2022_28801_MOESM3_ESM.pdf]

nature portfolio

Corresponding author(s): Claudia Capparelli
Andrew E. Aplin

Last updated by author(s): February 04, 2022

Reporting Summary

Nature Portfolio wishes to improve the reproducibility of the work that we publish. This form provides structure for consistency and transparency in reporting. For further information on Nature Portfolio policies, see our Editorial Policies and the Editorial Policy Checklist.

Statistics

For all statistical analyses, confirm that the following items are present in the figure legend, table legend, main text, or Methods section.

n/a	Confirmed	
☐	☒	The exact sample size (*n*) for each experimental group/condition, given as a discrete number and unit of measurement
☐	☒	A statement on whether measurements were taken from distinct samples or whether the same sample was measured repeatedly
☐	☒	The statistical test(s) used AND whether they are one- or two-sided<br>*Only common tests should be described solely by name; describe more complex techniques in the Methods section.*
☐	☒	A description of all covariates tested
☐	☒	A description of any assumptions or corrections, such as tests of normality and adjustment for multiple comparisons
☐	☒	A full description of the statistical parameters including central tendency (e.g. means) or other basic estimates (e.g. regression coefficient) AND variation (e.g. standard deviation) or associated estimates of uncertainty (e.g. confidence intervals)
☐	☒	For null hypothesis testing, the test statistic (e.g. *F*, *t*, *r*) with confidence intervals, effect sizes, degrees of freedom and *P* value noted<br>*Give P values as exact values whenever suitable.*
☒	☐	For Bayesian analysis, information on the choice of priors and Markov chain Monte Carlo settings
☐	☒	For hierarchical and complex designs, identification of the appropriate level for tests and full reporting of outcomes
☐	☒	Estimates of effect sizes (e.g. Cohen's *d*, Pearson's *r*), indicating how they were calculated

Our web collection on statistics for biologists contains articles on many of the points above.

Software and code

Policy information about availability of computer code

Data collection	The data generated in this paper consists of next-generation RNA sequencing data obtained through Illumina instruments as FASTQ files.<br><br>Raw reads from publicly available SRA RNA-seq datasets were downloaded using SRA toolkit (v 2.10.4)
Data analysis	- RNA-Seq datasets: STAR (v2.7.0d); RSEM (v1.2.28); DESeq2 (v1.28.1); GSEA (v3.0 and 4.0.1); iSeqQC (v1.0.0); GSVA (v1.40.1); R-Project (v 3.5.1 and 4.0.2 https://www.R-project.org/)<br><br>- scRNA-Seq cell culture dataset: SCopeLoomR (v0.10.2 https://github.com/aertslab/SCopeLoomR); edgeR (v 3.30.3); R-Project (v 4.0.2 https://www.R-project.org/)<br><br>- scRNA-Seq patient tumor dataset: Single Cell Portal (https://singlecell.broadinstitute.org/single_cell/study/SCP109/melanoma-immunotherapy-resistance#study-visualize)<br><br>- TCGA SKCM: cBioPortal v3.7.22(https://www.cbioportal.org/)<br><br>- visualization of RNA-Seq, scRNA-Seq cell culture and drug screen dataset: VennDiagram (v 1.6.20 https://CRAN.R-project.org/package=VennDiagram); ggplot2 (v 3.3.2 https://ggplot2.tidyverse.org/); pheatmap (v 1.0.12 https://CRAN.R-project.org/package=pheatmap)<br><br>IncuCyte experiments: R version 4.0.4 R Core Team. R: A Language and Environment for Statistical Computing. Vienna, Austria (2017). Available from: https://www.R-project.org/.<br><br>In vivo studies: SAS 9.4 (SAS Institute Inc., Cary, NC).

Flow cytometry: FlowJo (v10.6.1)

For manuscripts utilizing custom algorithms or software that are central to the research but not yet described in published literature, software must be made available to editors and reviewers. We strongly encourage code deposition in a community repository (e.g. GitHub). See the Nature Portfolio guidelines for submitting code & software for further information.

Data

Policy information about availability of data

All manuscripts must include a data availability statement. This statement should provide the following information, where applicable:

- Accession codes, unique identifiers, or web links for publicly available datasets
- A description of any restrictions on data availability
- For clinical datasets or third party data, please ensure that the statement adheres to our policy

RNA Seq data associated with this publication can be found under BioProject numbers:
PRJNA701949: MeWo parental and gSOX10 samples (https://www.ncbi.nlm.nih.gov/bioproject/PRJNA701949)
PRJNA748713: A375 parental, gSOX10 and combo-resistant tumor samples (https://www.ncbi.nlm.nih.gov/bioproject/PRJNA748713)
PRJNA748714: 1205 LuTR parental and PLX8394 resistant tumor samples (https://www.ncbi.nlm.nih.gov/bioproject/PRJNA748714)

Human and mouse reference genomes (GRCh38.p12 and GRCm38.p6) and gene & transcript annotation data (v28, v30 and M25) were obtained from GENCODE: https://www.gencodegenes.org/

Invasive and proliferative signatures were obtained from:
https://static-content.springer.com/esm/art%3A10.1038%2Fncomms7683/MediaObjects/41467_2015_BFncomms7683_MOESM1477_ESM.xlsx

GSEA Gene set collections (MSigDB Hallmark and GO Biological Process, v6.2 and v7.0) were obtained from:
https://www.gsea-msigdb.org/gsea/downloads.jsp

TCGA SKCM data originated from (cBioPortal v3.7.22):
http://gdac.broadinstitute.org/runs/stddata__2016_01_28/data/SKCM/20160128/

The following publicly available data were used in this study:
SRP029434: Patient tumors pre and post MAPK pathway inhibitor therapy, A375 parental and A375 shSOX10 samples. (https://www.ncbi.nlm.nih.gov/sra/SRP029434)
SRP215051: Six cell lines with SOX10 knockdown and matched control samples. (https://www.ncbi.nlm.nih.gov/sra/SRP215051)
SRP074198: 53 human melanoma cell line samples. (https://www.ncbi.nlm.nih.gov/sra/SRP074198)
SRP247646: Four mouse melanoma cell lines with four replicate samples for each. (https://www.ncbi.nlm.nih.gov/sra/SRP247646)

Human melanoma cell line annotation data were obtained from:
https://ars.els-cdn.com/content/image/1-s2.0-S1535610818301223-mmc2.xlsx

Single cell RNA Seq data for 7 treatment-naïve and 7 immune checkpoint inhibitor resistant melanoma patient tumors were originated from Jerby-Arnon, et al., 2018 (https://doi.org/10.1016/j.cell.2018.09.006). Cell annotation and normalized expression data are freely available for downloading in bulk from the Single Cell Portal (https://singlecell.broadinstitute.org/single_cell/study/SCP109/melanoma-immunotherapy-resistance) after registering for an account using a Google-managed identity (https://singlecell.broadinstitute.org/single_cell/terms_of_service).

Wouters_Human_Melanoma .loom file consisting of scRNA Seq data for 10 melanoma cultures were obtained from:
https://scope.aertslab.org/#/Wouters_Human_Melanoma/Wouters_Human_Melanoma%2F10_Baselines_filteredRegulons.loom/gene

Fig 1: Single Cell Portal
Fig 2: PRJNA701949 and PRJNA748713
Fig 5: SRP029434 and PRJNA748713
Fig 6: Wouters_Human_Melanoma

Supp Fig 1: PRJNA701949, PRJNA748713, SRP029434, and SRP215051
Supp Fig 4: SRP029434, PRJNA748713 and PRJNA748714
Supp Fig 5: Wouters_Human_Melanoma, TCGA SKCM, SRP247646 and SRP074198

Drug screen data associated with this publication can be found in the Source data file.

Field-specific reporting

Please select the one below that is the best fit for your research. If you are not sure, read the appropriate sections before making your selection.

☒ Life sciences ☐ Behavioural & social sciences ☐ Ecological, evolutionary & environmental sciences

For a reference copy of the document with all sections, see nature.com/documents/nr-reporting-summary-flat.pdf

Life sciences study design

All studies must disclose on these points even when the disclosure is negative.

Sample size	For mouse experiments, the sample size was established to provide 81% power to detect differences among 4 means, corresponding to the effect sizes of 0.5, 1.0, and 1.5 for comparison of the three treated groups vs. vehicle control, assuming a common standard deviation and using an F test with alpha=0.025.<br><br>For in vitro studies, the samples size (three biological replicates), was establish with the goal to have 80% power at a two-sided alpha level of 0.05 to detect a minimum difference of an effect size of about 2.75, in means in terms of standard deviation (SD) units, between different groups using two-sample t-test, which requires 3 samples per group.
Data exclusions	No data were excluded from any assay
Replication	For in vitro assays associated with statistical analysis: Cell growth assays (IncuCyte), 3D spheroid, and scratch wound assays were performed as 3 independent experiments (biological replicates) for rigor and to perform statistical analysis.<br>For all the experiments described within the manuscript all attempts of replication were successful. The number of replicates is indicated in each figure and/or legend.
Randomization	Mice harboring tumors with similar sizes were randomly divided into 4 different cohorts.<br>For in vitro experiments, samples were randomized in control and treatment groups, as indicated in the figures.
Blinding	For IHC analysis, sample labeling was available to the pathologist. However, the pathologist was not aware how the genetic alterations would have affected the tumor sample.<br>For in vitro experiments, blinding was impossible and there was no step that could have introduced personal bias.

Reporting for specific materials, systems and methods

We require information from authors about some types of materials, experimental systems and methods used in many studies. Here, indicate whether each material, system or method listed is relevant to your study. If you are not sure if a list item applies to your research, read the appropriate section before selecting a response.

Materials & experimental systems

n/a	Involved in the study	
☐	☒	Antibodies
☐	☒	Eukaryotic cell lines
☒	☐	Palaeontology and archaeology
☐	☒	Animals and other organisms
☐	☒	Human research participants
☒	☐	Clinical data
☒	☐	Dual use research of concern

Methods

n/a	Involved in the study	
☒	☐	ChIP-seq
☐	☒	Flow cytometry
☒	☐	MRI-based neuroimaging

Antibodies

Antibodies used	ErbB3 (#4754), PDGFR-beta (#3169), SOX10 (#89356), MITF (#12590), pS780-Rb (#9307), p21Cip1 (#2947), ZEB1 (#3396S), WNT5 (#2530), N-cadherin (#13116), HSP90 (#4877), pRB S807/811 (#9308), cIAP1 (#7065), cIAP2 (#3130), and XIAP (#2045) primary antibodies were purchased from Cell Signaling Technology. FN1 (# ab45688), SOX10 (#ab227680), Ki67 (#ab16667) and Collagen IV (#ab86042) primary antibodies were purchased from Abcam (Cambridge, MA). Actin (A2066) and FN1 (#F3648) primary antibodies were purchased from Sigma-Aldrich Co. Cyclin D3 (sc-182) primary antibody was purchased from Santa Cruz Biotechnology. p27Kip1 (#610241) and pFAK Y397 (#611806) primary antibodies were purchased from BD Transduction Laboratory. Goat Anti-Mouse IgG (#401215) and Goat Anti-Rabbit IgG (#401315) secondary antibodies were purchased from Sigma-Aldrich. HRP multimer cocktail secondary antibodies (#760-500) were purchased from Ventana. Biotinylated anti-rabbit (#BA-1000) secondary antibody was purchased from Vector Laboratories. AlexaFluor-488 (#A-11034), AlexaFluor-594 (#A-11032) and AlexaFluor-647 (#A-21236) secondary antibodies were purchased from Invitrogen.
Validation	The primary antibodies in this study have been validated by the manufacturer and have been used commonly in other publications. The details of validation for each antibody can be viewed on the vendor website links shown below:<br><br>ErbB3: https://www.cellsignal.com/products/primary-antibodies/her3-erbb3-1b2e-rabbit-mab/4754<br>PDGFRb: https://www.cellsignal.com/products/primary-antibodies/pdgf-receptor-b-28e1-rabbit-mab/3169<br>SOX10: https://www.cellsignal.com/products/primary-antibodies/sox10-d5v9l-rabbit-mab/89356<br>SOX10: https://www.abcam.com/sox10-antibody-sp267-ab227680.html<br>MITF: https://www.cellsignal.com/products/primary-antibodies/mitf-d5g7v-rabbit-mab/12590<br>pS780-Rb: https://www.cellsignal.com/products/primary-antibodies/phospho-rb-ser780-antibody/9307<br>p21Cip1: https://www.cellsignal.com/products/primary-antibodies/p21-waf1-cip1-12d1-rabbit-mab/2947

ZEB1: https://www.cellsignal.com/products/primary-antibodies/zeb1-d80d3-rabbit-mab/3396
WNT5: https://www.cellsignal.com/products/primary-antibodies/wnt5a-b-c27e8-rabbit-mab/2530
N-cadherin: https://www.cellsignal.com/products/primary-antibodies/n-cadherin-d4r1h-xp-rabbit-mab/13116
HSP90: https://www.cellsignal.com/products/primary-antibodies/hsp90-c45g5-rabbit-mab/4877
pRB S807/811: https://www.cellsignal.com/products/primary-antibodies/phospho-rb-ser807-811-antibody/9308
cIAP1:https://www.cellsignal.com/products/primary-antibodies/c-iap1-d5g9-rabbit-mab/7065
cIAP2: https://www.cellsignal.com/products/primary-antibodies/c-iap2-58c7-rabbit-mab/3130
XIAP: https://www.cellsignal.com/products/primary-antibodies/xiap-3b6-rabbit-mab/2045
FN1: https://www.abcam.com/fibronectin-antibody-f14-ab45688.html
FN1: https://www.sigmaaldrich.com/US/en/product/sigma/f3648
Ki67: https://www.abcam.com/ki67-antibody-sp6-ab16667.html
Collagen IV: https://www.abcam.com/collagen-iv-antibody-1043-ab86042.html
Actin: https://www.sigmaaldrich.com/US/en/product/sigma/a2066
Cyclin D3: https://www.scbt.com/p/cyclin-d3-antibody-d-7?gclid=CjwKCAiAz--OBhBIEiwAG1rIOkX4eXm7S5EY05Syah0-kd3BLJVDMkotiDQsQolwlIQ4isz31lXvLhoCpS4QAvD_BwE
p27Kip1: https://www.bdbiosciences.com/en-us/products/reagents/microscopy-imaging-reagents/immunofluorescence-reagents/purified-mouse-anti-p27-kip1.610241
pFAK Y397: https://www.bdbiosciences.com/en-eu/products/reagents/microscopy-imaging-reagents/immunofluorescence-reagents/purified-mouse-anti-human-fak-py397.611806

Eukaryotic cell lines

Policy information about cell lines

Cell line source(s)	MeWo cells were kindly donated by Dr. Barbara Bedogni, when at Case Western Reserve, Cleveland, OH in 2014, A375 parental cells were purchased from ATCC in 2005, 1205Lu, WM1366 and WM1361 were provided by Dr. Meenhard Herlyn, The Wistar Institute, Philadelphia, PA in 2005 and SKMEL28 cells were purchased from ATCC in 2002.
Authentication	Short-tandem repeat analysis was performed to authenticate cell lines. All cell lines matched known profiles.
Mycoplasma contamination	Cells were assayed for mycoplasma contamination every two months with MycoScope Kit (Genlantis). All cell lines used in our experiments were negative for mycoplasma contamination.
Commonly misidentified lines (See ICLAC register)	None

Animals and other organisms

Policy information about studies involving animals; ARRIVE guidelines recommended for reporting animal research

Laboratory animals	NOD.Cg-Prkdcscid Il2rgtm1Wjl/SzJ (NSG) mice male and female 6-8 weeks old.<br>NU/J, homozygous, female mice 6–8 weeks old.<br>All animals are provided with food and water ad libitum, and housed in cages (with a maximum of 5 mice/cage) in a temperature and humidity-controlled environment. Animals are maintained in housing conditions that allow for normal species behavior to minimize the development of abnormal behaviors, and have access to humane and veterinary care.<br>Mice were sacrificed when the tumor volume was greater than 1000 mm3.
Wild animals	The study did not involve wild animals
Field-collected samples	No field collected samples were used in the study
Ethics oversight	Animal experiments were performed at a Thomas Jefferson University facility that is accredited by the Association for the Assessment and Accreditation of Laboratory Animal Care. The Institutional Animal Care and Use Committee at Thomas Jefferson University approved these studies (Protocol #: 01052)

Note that full information on the approval of the study protocol must also be provided in the manuscript.

Human research participants

Policy information about studies involving human research participants

Population characteristics	Human Samples were collected at Thomas Jefferson Hospital and no further information associated with the samples was provided.
Recruitment	No recruitment was required for our study
Ethics oversight	Institutional Review Board at Thomas Jefferson University (#10D.341)

Note that full information on the approval of the study protocol must also be provided in the manuscript.

Flow Cytometry

Plots

Confirm that:

- [x] The axis labels state the marker and fluorochrome used (e.g. CD4-FITC).
- [x] The axis scales are clearly visible. Include numbers along axes only for bottom left plot of group (a 'group' is an analysis of identical markers).
- [x] All plots are contour plots with outliers or pseudocolor plots.
- [x] A numerical value for number of cells or percentage (with statistics) is provided.

Methodology

Sample preparation	300,000 cells per cell line were plated and left to incubate overnight. The next day, cells were trypsinized, spun down, washed twice with PBS and resuspended in PBS with 1% FBS. Cells were strained through a 70um cell strainer and transferred to flow tubes.
Instrument	BD FACSCelesta
Software	FlowJo
Cell population abundance	10,000 events were collected per sample
Gating strategy	FSC-A/SSC-A plots were used to gate out cell debris. The cells were subsequently gated on FSC-A/FSC-H to exclude doublets, and the resulting population was assessed for GFP and mCherry expression.

- [x] Tick this box to confirm that a figure exemplifying the gating strategy is provided in the Supplementary Information.
