# Supplementary material for: Targeting SOX10-deficient cells to reduce the dormant-invasive phenotype state in melanoma
Source: Nat Commun. 2022 Mar 16;13:1381. doi: 10.1038/s41467-022-28801-y (PMC8927161; doi:10.1038/s41467-022-28801-y)
Supplement: Supplementary file 4 — Source data [file 41467_2022_28801_MOESM4_ESM.zip › Source Data/full_blots_NC_Final_Revision.pptx]

## Slide 1
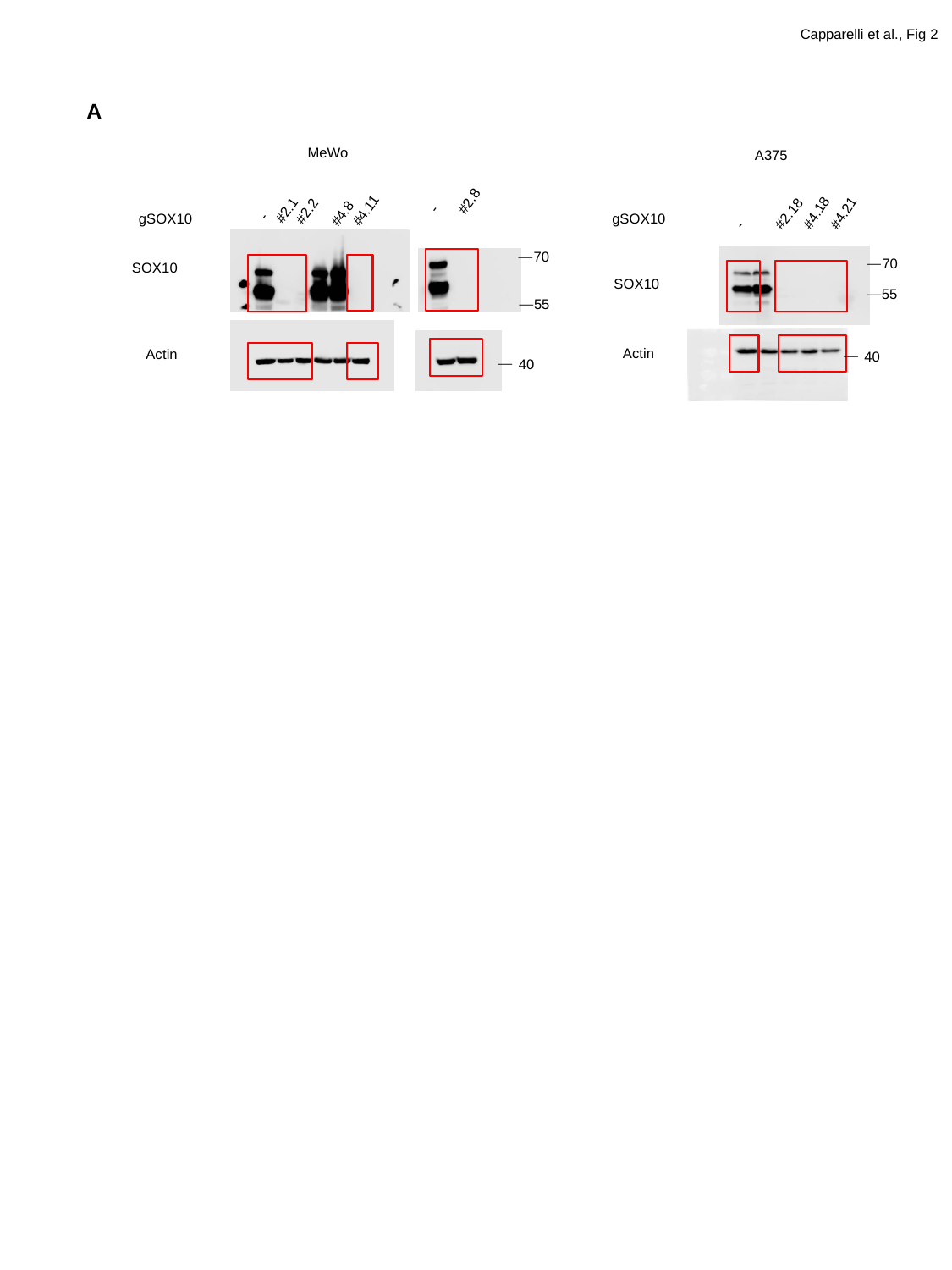

Capparelli et al., Fig 2
A
MeWo
-
#2.1
#2.2
#4.8
#4.11
gSOX10
SOX10
Actin
A375
#4.18
#4.21
-
#2.18
gSOX10
SOX10
Actin
-
#2.8
70
⎯
70
⎯
55
⎯
55
⎯
⎯
40
⎯
40

## Slide 2
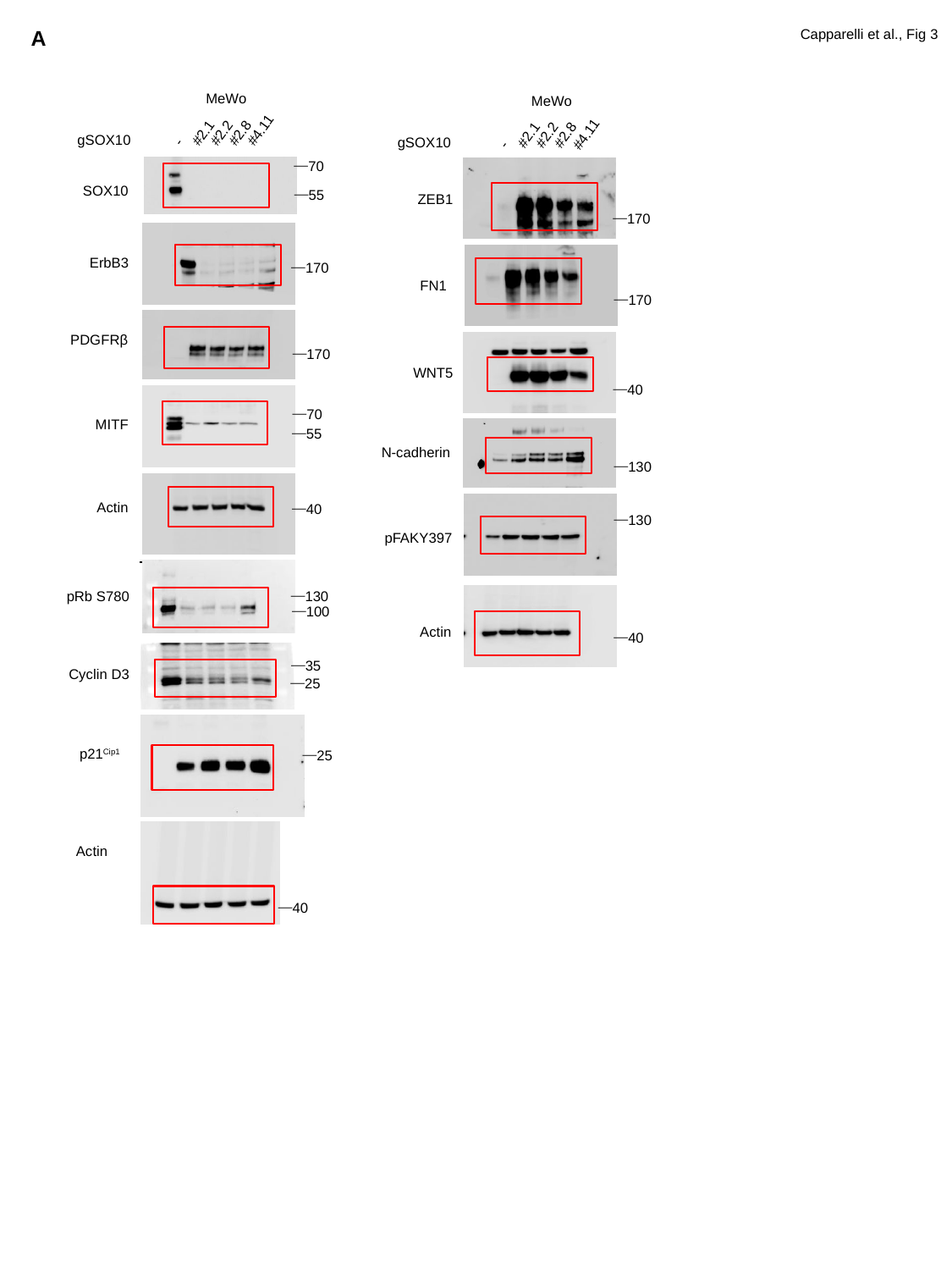

A
Capparelli et al., Fig 3
MeWo
-
#2.1
#2.2
#2.8
#4.11
gSOX10
ZEB1
FN1
WNT5
N-cadherin
pFAKY397
Actin
MeWo
-
#2.1
#2.2
#2.8
#4.11
gSOX10
⎯70
SOX10
⎯55
⎯170
ErbB3
⎯170
⎯170
PDGFRβ
⎯170
⎯40
⎯70
MITF
⎯55
⎯130
Actin
⎯40
⎯130
pRb S780
⎯130
⎯100
⎯40
⎯35
Cyclin D3
⎯25
p21Cip1
⎯25
Actin
⎯40

## Slide 3
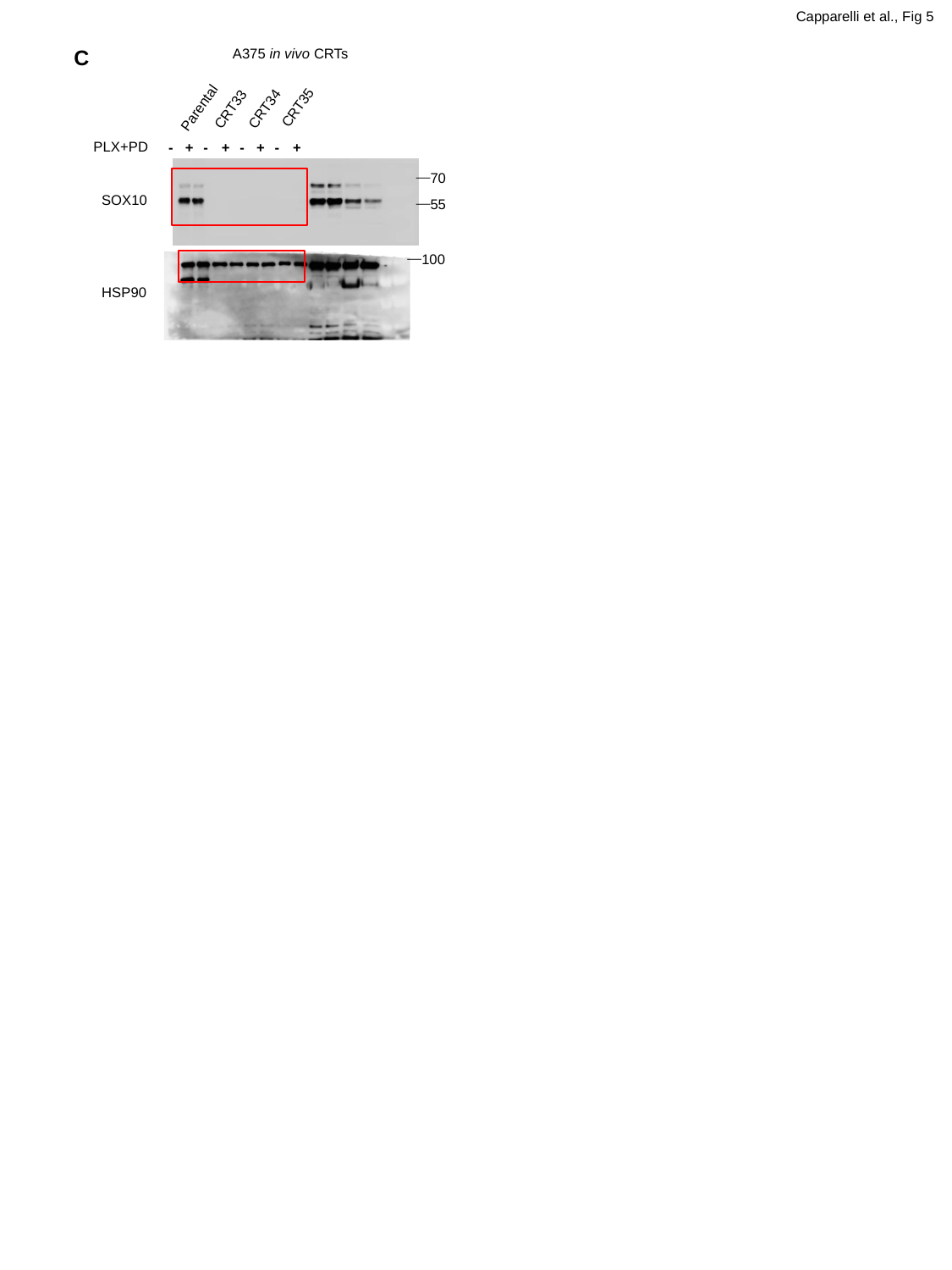

Capparelli et al., Fig 5
C
A375 in vivo CRTs
CRT35
CRT34
Parental
CRT33
PLX+PD
-
+
-
+
-
+
-
+
⎯70
SOX10
⎯55
⎯100
HSP90

## Slide 4
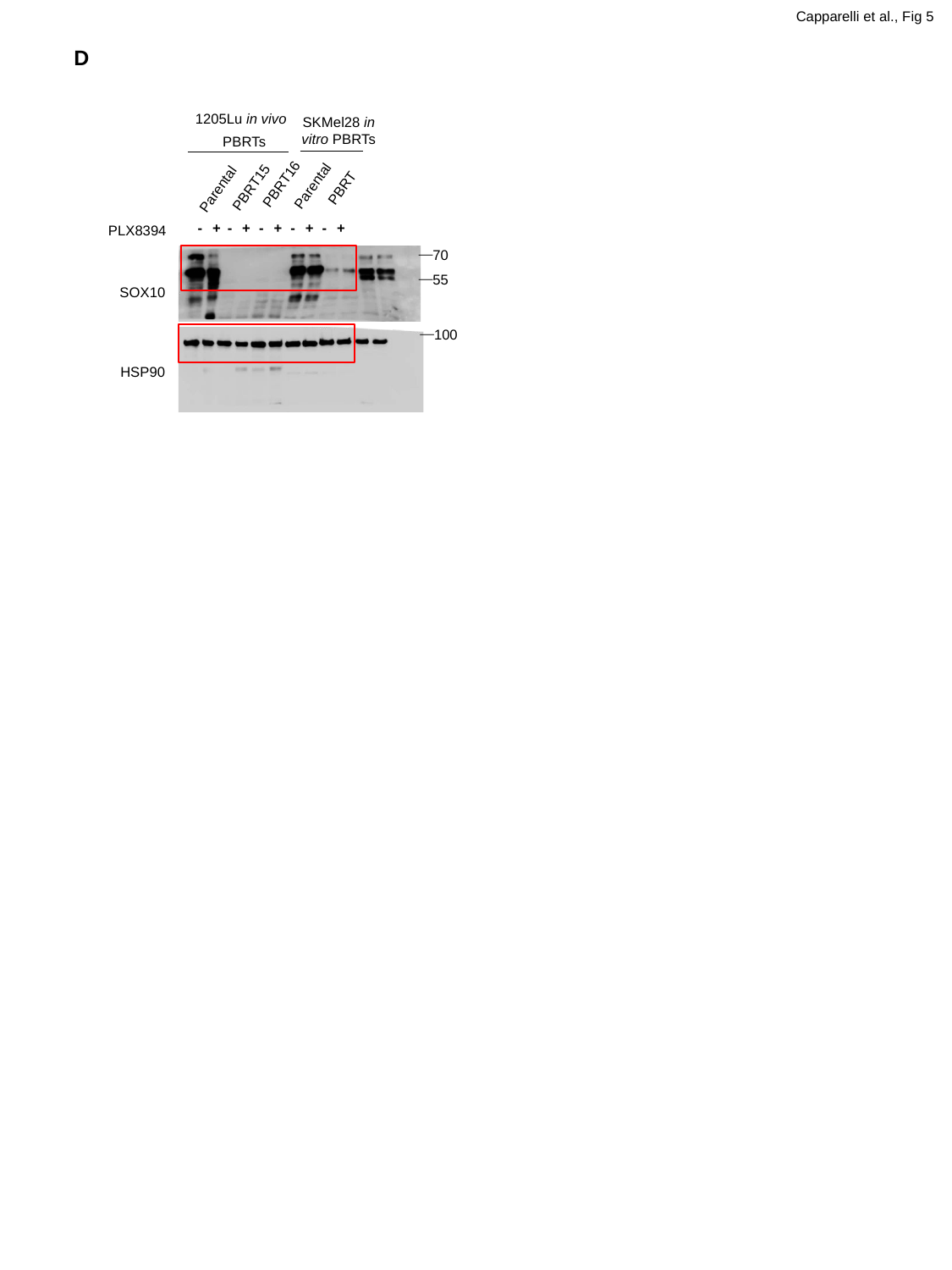

Capparelli et al., Fig 5
D
1205Lu in vivo
SKMel28 in vitro PBRTs
PBRTs
PBRT
PBRT16
Parental
PBRT15
Parental
-
+
-
+
-
+
-
+
-
+
PLX8394
⎯70
⎯55
SOX10
⎯100
HSP90

## Slide 5
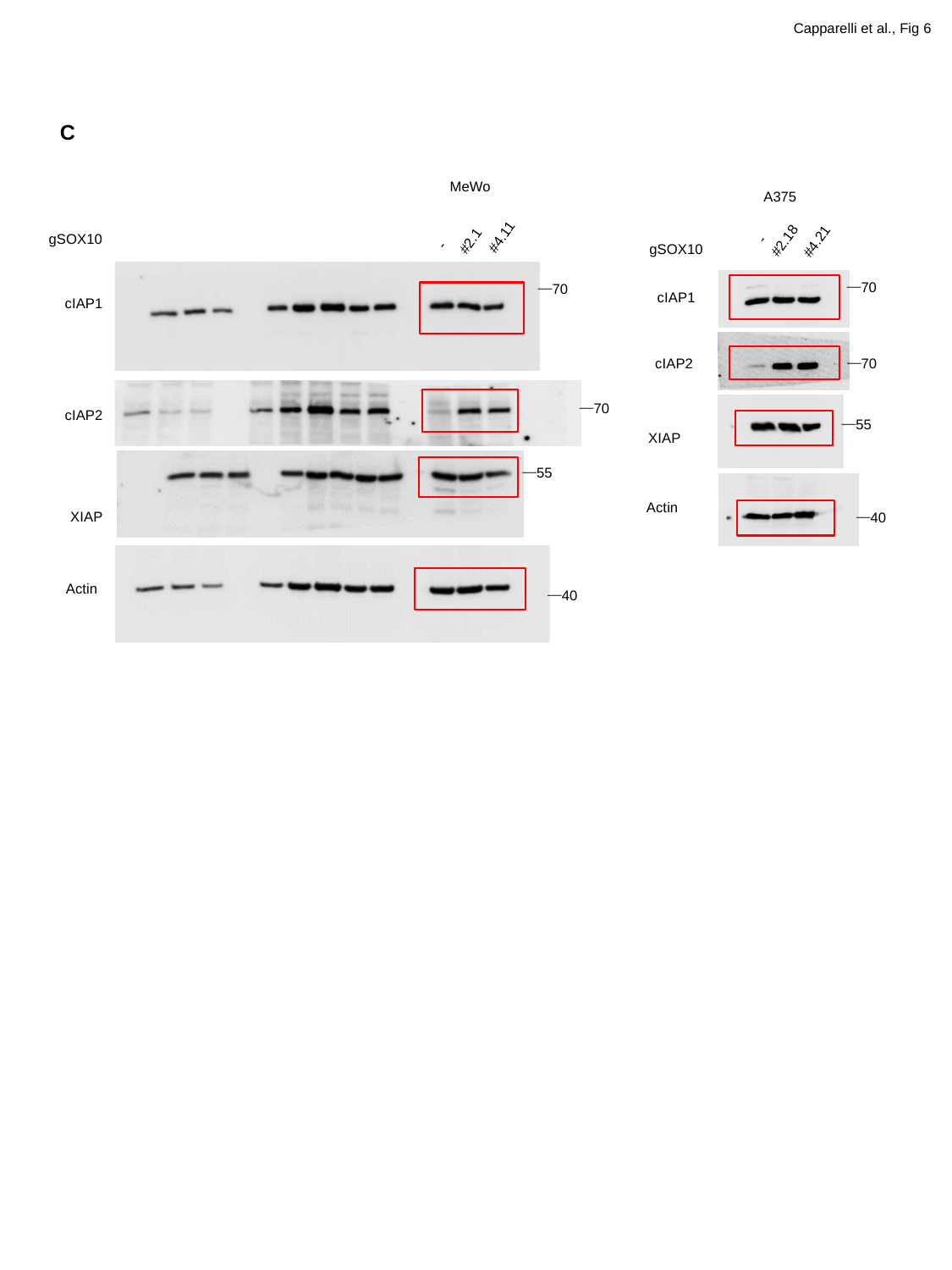

Capparelli et al., Fig 6
C
MeWo
A375
-
#2.18
#4.21
gSOX10
cIAP1
cIAP2
XIAP
Actin
-
#4.11
#2.1
gSOX10
⎯70
⎯70
cIAP1
⎯70
⎯70
cIAP2
⎯55
⎯55
XIAP
⎯40
Actin
⎯40

## Slide 6
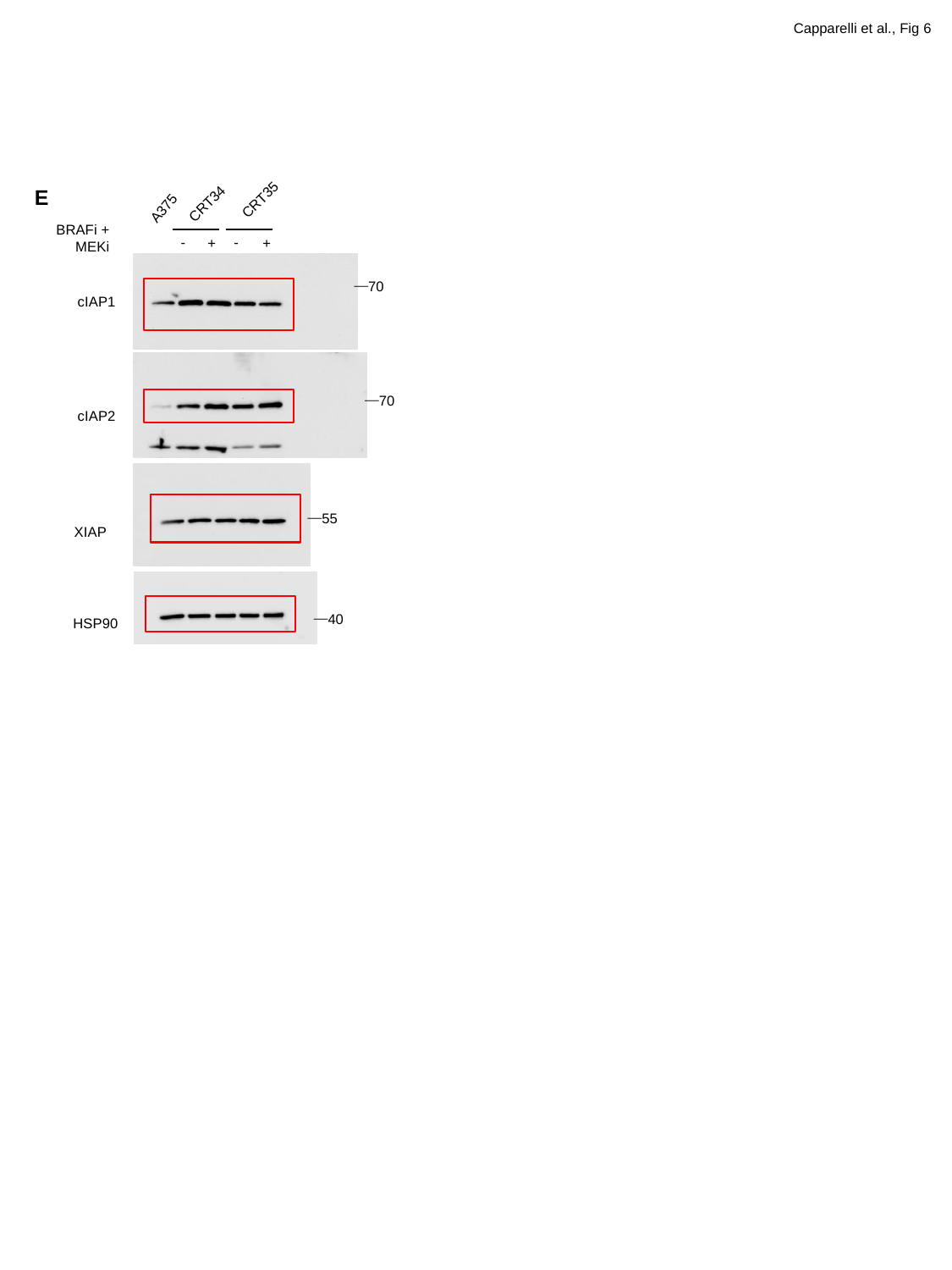

Capparelli et al., Fig 6
E
 CRT35
A375
 CRT34
BRAFi + MEKi
-
-
+
+
⎯70
cIAP1
⎯70
cIAP2
⎯55
XIAP
⎯40
HSP90
